# Nanotubes from the Misfit Layered Compound (SmS)_1.19_TaS_2_: Atomic Structure, Charge Transfer, and Electrical Properties

**DOI:** 10.1021/acs.chemmater.1c04106

**Published:** 2022-02-10

**Authors:** M. B. Sreedhara, Kristýna Bukvišová, Azat Khadiev, Daniel Citterberg, Hagai Cohen, Viktor Balema, Arjun K. Pathak, Dmitri Novikov, Gregory Leitus, Ifat Kaplan-Ashiri, Miroslav Kolíbal, Andrey N. Enyashin, Lothar Houben, Reshef Tenne

**Affiliations:** † Department of Molecular Chemistry and Materials Science, Weizmann Institute of Science, Rehovot 7610001, Israel; ‡ CEITEC − Central European Institute of Technology, Brno University of Technology, Purkyňova 123, 612 00 Brno, Czech Republic; § Deutsches Elektronen-Synchrotron DESY, Notkestr. 85, 22607 Hamburg, Germany; ∥ Department of Chemical Research Support, Weizmann Institute, Rehovot 7610001, Israel; ⊥ Ames Laboratory, U.S. Department of Energy, Ames, Iowa 50011-3020, United States; # ProChem, Inc., 826 Roosevelt Road, Rockford, Illinois 61109, United States; ¶ Department of Physics, SUNY Buffalo State, Buffalo, New York 14222, United States; ∇ Institute of Physical Engineering, Brno University of Technology, Technická 2, 616 69 Brno, Czech Republic; ○ Institute of Solid State Chemistry UB RAS, 620990 Ekaterinburg, Russian Federation; ◆ Institute of Natural Sciences and Mathematics, Ural Federal University, 620083 Ekaterinburg, Russian Federation

## Abstract

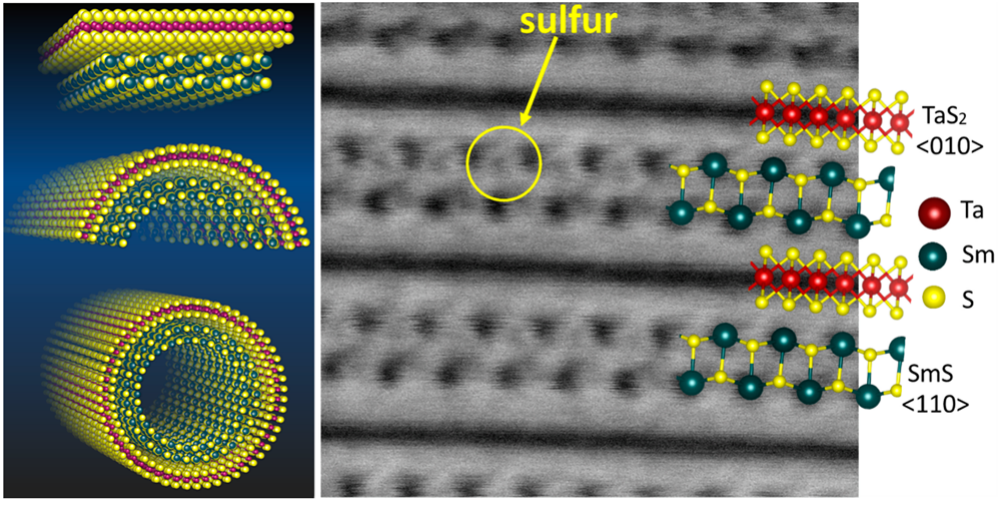

Misfit layered compounds (MLCs) MX-TX_2_, where M, T = metal atoms and X = S, Se, or Te, and their nanotubes are of significant interest due to their rich chemistry and unique quasi-1D structure. In particular, LnX-TX_2_ (Ln = rare-earth atom) constitute a relatively large family of MLCs, from which nanotubes have been synthesized. The properties of MLCs can be tuned by the chemical and structural interplay between LnX and TX_2_ sublayers and alloying of each of the Ln, T, and X elements. In order to engineer them to gain desirable performance, a detailed understanding of their complex structure is indispensable. MLC nanotubes are a relative newcomer and offer new opportunities. In particular, like WS_2_ nanotubes before, the confinement of the free carriers in these quasi-1D nanostructures and their chiral nature offer intriguing physical behavior. High-resolution transmission electron microscopy in conjunction with a focused ion beam are engaged to study SmS-TaS_2_ nanotubes and their cross-sections at the atomic scale. The atomic resolution images distinctly reveal that Ta is in trigonal prismatic coordination with S atoms in a hexagonal structure. Furthermore, the position of the sulfur atoms in both the SmS and the TaS_2_ sublattices is revealed. X-ray photoelectron spectroscopy, electron energy loss spectroscopy, and X-ray absorption spectroscopy are carried out. These analyses conclude that charge transfer from the Sm to the Ta atoms leads to filling of the Ta 5*d*_*z*^2^_ level, which is confirmed by density functional theory (DFT) calculations. Transport measurements show that the nanotubes are semimetallic with resistivities in the range of 10^–4^ Ω·cm at room temperature, and magnetic susceptibility measurements show a superconducting transition at 4 K.

## Introduction

**Figure 1:**
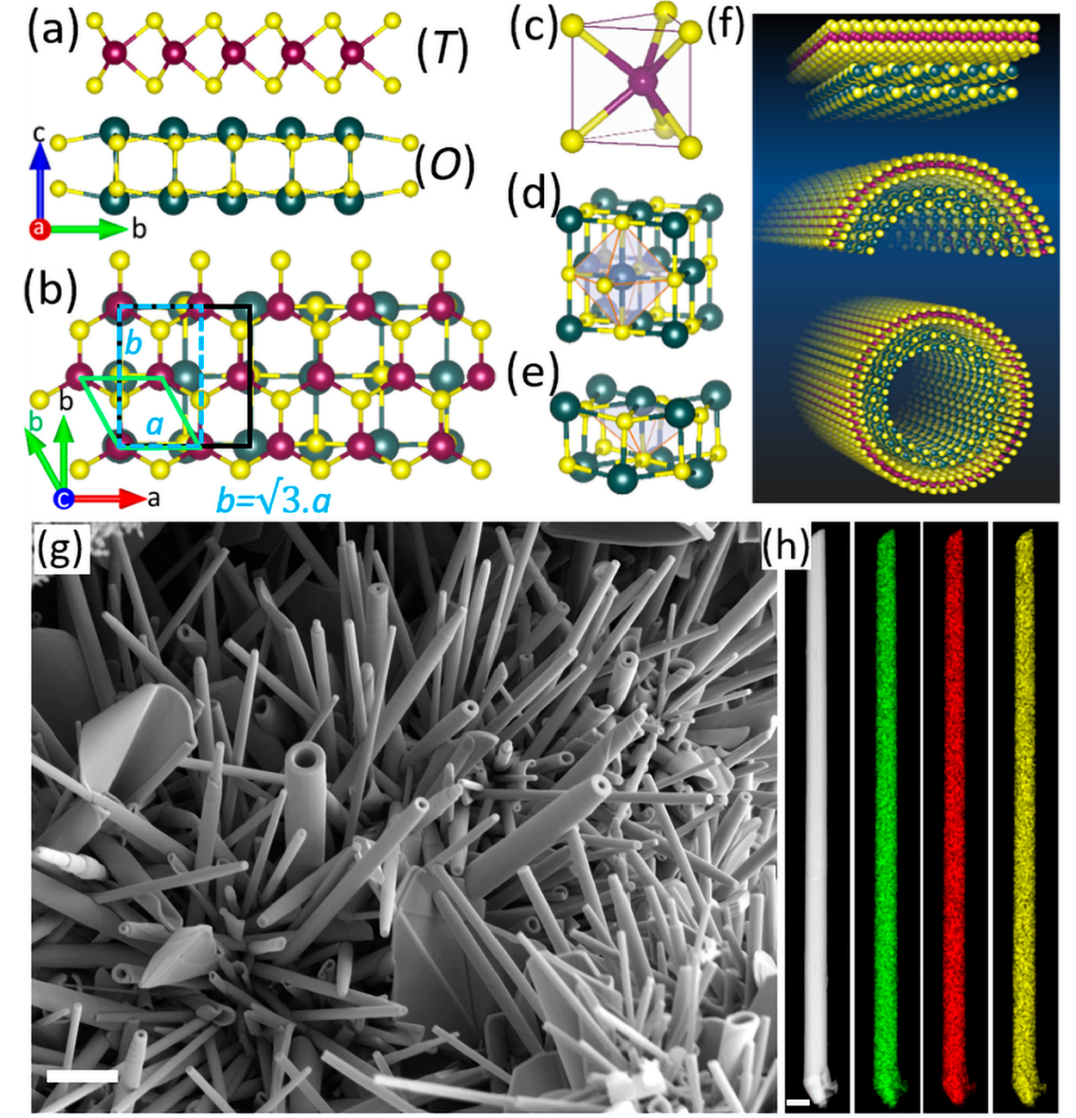
Structure, morphology, and chemical analyses of misfit (SmS)_1.19_TaS_2_. Schematic drawing of the MLC lattice structure projected (a) in the *a*–*b* plane and (b) along the *c* direction, respectively. In the *a*–*b* plane, the incommensurate-*a* and commensurate-*b* crystallographic axes of the orthohexagonal unit cell (*b* = √3*a*) are marked in cyan color. The *O* and *T* represent orthorhombic and trigonal prismatic coordination of Sm in the rocksalt unit and Ta in the hexagonal unit and are graphically represented in (c) and (d), respectively. The single-layer MLC slab constitutes half a unit cell of rocksalt structure and is graphically shown in (e) with corresponding coordination of Sm. (f) Graphical rendering of the formation mechanism of an MLC nanotube via misfit strain relaxation (folding) and seaming of the rim atoms. (g) SEM image of (SmS)_1.19_TaS_2_ flakes and nanotubes obtained by quenching high-temperature CVT reaction to ambient conditions; scale bar is 2 μm. (h) STEM HAADF images of a single (SmS)_1.19_TaS_2_ MLC nanotube and corresponding SEM-EDS chemical maps (Sm, green; Ta, red; and S, yellow); scale bar is 200 nm.

Misfit layered compounds (MLCs) are a class of two-dimensional (2D) materials receiving considerable attention due to their unique structure, crystallographic diversity, and chemically tailorable characteristics (*vide infra*).^1−8^ Among the MLCs, the chalcogenide-based MLCs are of special interest due to their metallic and semiconducting properties. The chalcogenide-based MLCs with the general formula (MX)_(1+*y*)*m*_(TX_2_)_*n*_ (where M = Sn, Sb, Pb, Bi, Ln rare-earth atom, Y; T = Ta, Nb, V, Cr; and X is a chalcogen atom S, Se, Te) constitute a superstructure of alternating slabs of distorted rocksalt MX and hexagonal TX_2_ structural units (see Figure 1a–d). For the most common case, it is abbreviated as MX-TX_2_ (*m* = *n* = 1). Another shortened notation for MLC often used in the literature is (*O*–*T*), indicating orthorhombic (O = MX) and trigonal prismatic (T = TX_2_) coordination, respectively.

With their unique structure and tunable properties, chalcogenide-based MLCs offer potential applications in thermoelectrics.^9−13^ In order to tune the desired character of MLCs such as electronic conductivity or phonon scattering and improve their thermoelectric performance, a detailed structural understanding of each sublayer (MX and TX_2_) atom by atom is highly desirable. Further, the periodic modulation due to nonstoichiometry and misfit strain can create strain waves and lead to vacancies in the structure (especially in the rocksalt unit), which may induce Anderson localization in these compounds.^14^ Advancement in sub-ångström resolution electron microscopy and growth techniques^15,16^ in recent times has prompted research into their local structure, charge transfer characteristics, and spectroscopic properties. The inception of misfit nanotubes over the past decade brought about another twist to their study.^17−19^

Due to their 1D structure and chiral nature, nanotubes of inorganic layered compounds, like WS_2_, offer intriguing physical properties,^20,21^ making the study of MLC nanotubes also highly warranted. MLC nanotubes of the kind SnS-SnS_2_ were first obtained serendipitously by laser ablation of SnS_2_ powder.^22^ Later on, rare-earth-based LnS-TaS_2_ MLCs and their nanotubes were studied quite extensively.^17,23−27^ LnS-TaS_2_ MLCs constitute a large family of compounds and are rather interesting owing to the significant charge transfer from the LnS unit to TaS_2_. The unique optical and magnetic properties of rare-earth compounds offer numerous potential applications.

Among the LnS-TaS_2_ family, SmS-TaS_2_ is of special interest due to the exotic physical properties of their binary sulfide constituents (*vide infra*).^7,8,28,29^ The binary SmS shows switching behavior, i.e., an ability to undergo reversible pressure-induced (at 6.5 kbar) semiconductor-to-metal transition at room temperature, which can be switched back to its original phase upon heating.^28,30−32^ As a result, Sm can accommodate two ground states configurations, either nonmagnetic Sm^2+^ (semiconducting SmS) with 4*f*^6^ (^7^F_0_) configuration or magnetic Sm^3+^ (metallic SmS) with 4*f*^5^ (^6^H_5/2_) configuration.^33^ Interestingly, in addition to the properties of SmS, the abundant electron coupling interactions in the 2H-TaS_2_ lead to compelling physical phenomena such as layer-dependent charge density waves^29^ and interfacial superconductivity.^34^ The combination of these physical properties from the respective binary phases and the atomic-scale manipulation of charge transfer in (SmS)_1.19_TaS_2_ can tune the electronic quantum phases, which could result in new physical properties. The binary SmS crystallizes in a rocksalt structure with the space group *Fm*3̅*m* (*a* = 5.97 Å, for Sm^2+^, and *a* = 5.57 Å, for the high-pressure phase, Sm^3+^), where Sm atoms are octahedrally coordinated to S atoms. 2H-TaS_2_ is a layered compound with hexagonal lattice (*a* = 3.314 Å, *c* = 12.097 Å, *P*6_3_/*mmc*), in which each Ta atom is bound to six sulfur atoms in a trigonal prismatic configuration.^35^ The structural motifs of SmS-TaS_2_ can be described in the orthorhombic space group *Fm*2*m* with *FF* centering,^7^ in which SmS and TaS_2_ are stacked layer by layer periodically along the *c*-axis (*a* = 3.29 Å, *b* = 5.67 Å, and *c* = 22.50 Å). Given the unit-cell dimensions of each sublattice, the misfit ratio *y* of the compound (SmS)_1+*y*_TaS_2_ can be found from the formula 1 + *y* = 2*a*_TaS2_/*a*_SmS_ to be 1.19. This factor represents also the deviation from the stoichiometry of the two subunits in the MLC. Previously, the SmS-TaS_2_ MLC was investigated using scanning tunneling micro(spectro)scopy, and it was concluded that the outermost layer on the surface of the cleaved crystal is SmS.^36^ On the other hand, high-resolution transmission electron microscopy (HRTEM) revealed that the outermost layer of MLC is the TaS_2_ layer.^37^ Also, the (SmS)_1.19_TaS_2_ surface was found to be semimetallic.^36,38^

The charge transfer from LnS to TaS_2_ stabilizes MLC structures and alters the electronic properties of the two subunits, appreciably.^39^ The amount of charge transfer can be tuned by alloying the rocksalt (LnS) unit with other rare earth or heteroatoms.^40,41^ The role of the charge transfer from the Ln atoms to the Ta atoms has been elucidated.^25,41^ In particular, since the work function of the LnS subunit is smaller than that of the hexagonal TaS_2_, charge transfer occurs from the rare-earth atom to the partially occupied 5*d*_*z*^2^_ level of the tantalum atom. This charge transfer modifies the effective valence state of the rare-earth atom (2+) closer to the more stable 3+ state. Furthermore, the 5*d*_*z*^2^_ level of the Ta atom, which dominates the density of states (DOS) at the Fermi level (*E*_f_), is getting almost filled by the charge transfer.^39,42^ This effect leads to an increase in the density of occupied states at *E*_f_ and reduces the hole conductivity. The charge transfer also stabilizes the 2H (trigonal prismatic coordination) polytype of TaS_2_, preventing its transformation into 1T with octahedral coordination and the ensuing charge density wave transition. This conjecture was supported by Raman spectroscopy of individual nanotubes.^27^ Nonetheless, direct evidence from transmission electron microscopy with the sub-ångström resolution was missing so far and is presented here.

In the present work, nanotubes (and flakes) of the misfit compound (SmS)_1.19_TaS_2_ were studied in detail to address the structural aspects atom by atom and correlate it with its properties. High-resolution scanning transmission electron microscopy (HR-STEM) with sub-ångström resolution was employed here to elucidate the atomic arrangements in the lattice of (SmS)_1.19_TaS_2_ nanotubes, which was not available before. Moreover, by using dual-beam focused ion beam (FIB) microscopy, lamellae of such nanotubes were prepared to enable direct imaging of the superstructure from the axial *b* direction (nanotube growth axis). This analysis yields the lattice structure of such MLC nanotubes in unprecedented detail and provides a pathway to correlate the structure with the physical properties of such 1D nanostructures.

X-ray photoelectron spectroscopy (XPS) and high-resolution electron energy loss spectroscopy (HR-EELS) were employed here to elucidate the core levels and valence band structure of the (SmS)_1.19_TaS_2_ MLC nanotubes and flakes and pure 2H-TaS_2_. It was shown that the 5*d*_*z*^2^_ level of Ta in MLC is getting filled up upon coupling with SmS and the charge transfer from Sm → Ta. The filling of Ta 5*d*_*z*^2^_ and valence conversion of Sm^2+^ to Sm^3+^ upon charge transfer was further confirmed by X-ray absorption (XAS) studies. These observations were validated with DFT calculations. Magnetic susceptibility measurements at low temperature show that (SmS)_1.19_TaS_2_ is superconducting below 5 K while the four-probe electrical measurements at room temperature show a semimetallic behavior of the nanotubes.

## Experimental Details

### Synthesis of (SmS)_1.19_TaS_2_ Misfit Nanotubes

Misfit nanotubes of (SmS)_1.19_TaS_2_ were prepared in evacuated quartz ampules by well-established chemical vapor transport (CVT) protocol. All the reactants were handled under the inert atmosphere in a glovebox to prevent oxidation. In a typical synthesis, stoichiometric amounts of Sm (Strem Chemicals 99.9%), Ta (Alfa Aesar 99.9%), and S (Sigma-Aldrich 99.98%) powders in the proportion of 1:1:3 (20.7 mg (0.13 mmol) of Sm; 25 mg (0.13 mmol) of Ta; and 13.2 mg (0.41 mmol) of S) were ground in an agate mortar. A catalytic amount of TaCl_5_ (3 mg, Sigma-Aldrich 99.99%) was used as a transport agent. The ampule was connected to a vacuum system and evacuated using a diffusion pump protected by a liquid nitrogen trap and backed with a rotary pump. The quartz ampules were sealed under vacuum (<1 × 10^–5^ Torr) and transferred to a preheated vertical furnace for the annealing process. The annealing was performed in two steps using two opposite temperature gradients under constant monitoring of the temperature inside the furnace using an external thermocouple. In the first step, the ampules were submitted to a thermal gradient of 400 °C at the bottom edge and 800 °C at the upper edge. After 1 h, the ampules were moved inside the furnace and exposed to an opposite temperature gradient between 825 °C at the bottom part and 400 °C at the upper part. After high-temperature annealing, the ampules were withdrawn from the furnace and were allowed to cool down to room temperature in ambient air. As previously observed, the mass transport to the colder edge was negligible and the products were accumulated in the high-temperature edge of the ampule. The product was collected and stored in a glovebox for further analysis. The materials have been synthesized several times to reproduce the growth, and the growth of misfit nanotubes and flakes was found to be very reproducible. The characterization details are given in the Supporting Information (SI).

### Computational Details

Spin-polarized DFT calculations in periodic boundary conditions were performed using the SIESTA 4.1 package.^43,44^ The core electrons were treated within the frozen core approximation, applying norm-conserving Troullier–Martins pseudopotentials. The only valence shells accounted for were for Ta and S, while the 5*p* shell was added as a semicore one for Sm. The single-ζ basis set was used for the description of valence orbitals. The *k*-point mesh was generated by the method of Monkhorst and Pack with a cutoff of 15 Å used for *k*-point sampling.^45^ The real-space grid used for the numerical integrations was set to correspond to the energy cutoff of 300 Ry. For geometry optimization, the exchange-correlation potential was described in Generalized Gradient Approximation (GGA) with the Perdew–Burke–Ernzerhof (PBE) parametrization. The calculations were performed using variable-cell and atomic position relaxations, with convergence criteria corresponding to the maximum residual stress of 0.1 GPa for each component of the stress tensor and the maximum residual force component of 0.05 eV/Å. The chosen DFT GGA protocol yields equilibrium lattice parameters for binary fcc-SmS (*a* = 5.97 Å) and 2H-TaS_2_ compounds (*a* = 3.35 Å, *c* = 11.62 Å) in fair agreement with the experimental data.

Classical GGA functionals are valuable for the description of structural and cohesion properties.^46^ Yet, they can underestimate the band gap and have an imperfect description of *d*- and *f*-electron correlations, tending to result in too delocalized spin density moments.^47,48^ Therefore, additional analysis of electronic structure for the geometry optimized crystal lattices was performed using the simplified rotationally invariant spin-polarized LDA+U formulation of DFT^49^ with an effective Coulomb repulsion parameter *U*_eff_ = 4 eV. Noteworthy, the GGA+U approach had led to a severely overestimated value of the band gap of SmS; hence, it was not employed any further.

Anisotropic dielectric functions were derived from a pseudopotential SIESTA DFT code, which was capable of addressing larger supercells of 50–100 atoms. Good qualitative agreement was found among SIESTA, the more accurate DFT, and the values of the anisotropic dielectric response of TaS_2_ in the literature. Nevertheless, minor quantitative discrepancies were observed, owing to the semicore orbitals, which are approximated by pseudopotentials.

### Magnetic Measurements

Magnetic measurements (both ac susceptibility and dc magnetization) of (SmS)_1.19_TaS_2_ were carried out by a superconducting quantum interference device (SQUID) and a physical property measurement system (PPMS, Quantum Design), in the temperature range 2–50 K and magnetic field up to 10 kOe. The ac susceptibility was measured at frequencies 1, 100, and 1000 Hz and a zero dc magnetic field. The samples were kept in a sealed quartz ampule under vacuum to prevent any deterioration before the magnetic measurements. The sample was loaded for magnetic measurements in the glovebox in an inert atmosphere. Similar samples were analyzed via XRD confirming that the impurities’ contents, like pure Ta and TaS_2_, are negligible. The samples were also measured using a magnetic property measurement system (MPMS-3). The dc magnetic moments were measured by VSM (vibrating sample magnetometry) mode in the temperature range from 2 to 300 K.

### Electrical Characterization

The nanotubes were dispersed in isopropanol and dripped onto a Si wafer with a ∼20 nm HfO_2_/∼270 nm SiO_2_ oxide stack on top. Selected nanotubes were contacted by electron beam lithography, utilizing an AR-P 679.04 (PMMA) resist (∼800 nm thick). For the electrical contacts, Cu (∼470 nm) and Au (∼20 nm) layers were evaporated using Ti (∼10 nm) as an adhesion layer (total thickness ∼ 500 nm) onto the developed sample, followed by a lift-off process. The electrical characterization was done at room temperature and at atmospheric pressure utilizing a Cascade Microtech MPS 150 probe station and Keithley 4200-SCS parameter analyzer. The area of the nanotube cross-section used for resistivity calculation was acquired from the SEM image assuming the cross section to be circular and fully filled with material. Note that, for the nanotube, the cross-sectional area is smaller, and hence, the resistivity calculated in this way is an upper bound value. The correction for a tubular cross-section yields resistivity values smaller by 20% at maximum. As for the length of the channel used in the calculations, the distance in between the inner contacts was used, which again resulted in a slight overestimation of the resistivity.

## Results and Discussion

As illustrated in Figure 1a,e, the rocksalt SmS structure in the MLC is modulated compared to pristine SmS (Figure 1d), whereas the hexagonal TaS_2_ is almost undistorted, resembling bulk 2H-TaS_2_. The SmS slab is made of only half a unit cell (along the *c*-axis) between the two TaS_2_ units of MLC. The Sm is coordinated to only five sulfur atoms (instead of six in the binary SmS) within the unit (Figure 1e) and consequently may have a strong dipolar interaction with sulfur atoms of the adjacent TaS_2_. MLC compounds tend to roll into nanotubes due to the misfit strain (between the rocksalt MX and the hexagonal TX_2_ layers) and seaming of the dangling bonds at the rim atoms.^50^ The folding mechanism is schematically depicted in Figure 1f. The MLC nanotubes of (SmS)_1.19_TaS_2_ were produced by a well-established chemical vapor transport protocol with slight modification in growth temperature to improve the yield. SEM micrographs of as-obtained (SmS)_1.19_TaS_2_ powder displayed in Figures 1h and S1a show nanotubular structures as a major product. Other common byproducts, such as flakes and nanoscrolls with similar chemical compositions (as confirmed by SEM-EDS) were also observed. Semiquantitative EDS analysis showed that the stoichiometry of the nanotubes is (SmS)_1.05_TaS_2_. The majority of the nanotubes display a constant diameter along the tube axis, whereas few of them showed a telescopic contour with varying diameters along the tube axis. SEM analysis revealed that the MLC tubes are grown perfectly under the established reaction conditions. It will be interesting to know what thermodynamic and kinetic factors could lead to either type of morphology such as nanotube/nanoscroll, but none of the *ex-situ* measurements could reveal those conditions. Statistical analysis of the nanotube size distribution was carried out using SEM images. This analysis revealed that the nanotubes display varying lengths, the majority of them falling within the range of 100–200 nm in diameter. Figure 1h shows low magnification STEM and STEM-energy-dispersive X-ray spectroscopy (EDS) chemical maps of the single (SmS)_1.19_TaS_2_ nanotube (see also Figure S1b). The nanotube is 200 nm in diameter, and the size of the tube is uniform along its entire length. The chemical maps reveal the uniform distribution of samarium (red), tantalum (green), and sulfur (yellow) elements throughout the tube. Quantitative STEM-EDS analysis of several nanotubes and flakes (Figure S1c,d) shows the stoichiometries (SmS)_1.08_TaS_2_ and (SmS)_1.05_TaS_2_, respectively, which are quite comparable to the theoretical value 1 + *y* = 1.19. Deviations are attributed to the experimental error as well as structural defects (*vide infr*a). No signal, which could be associated with the oxidation of the nanotube core, was obtained. A thin and somewhat nonuniform surface oxide layer (<1 nm) was occasionally observed.

X-ray diffraction (XRD) of (SmS)_1.19_TaS_2_ powder exhibits a strong diffraction pattern with a highly preferred orientation along the *c*-direction (see Figure S2). The observed patterns are consistent with an earlier report,^7^ and the interlayer spacing calculated from the (002) periodicity is 11.3 Å, equivalent to *c*/2 of the *FF* centered (SmS)_1.19_TaS_2_ misfit lattice. The strongly preferred orientations along the ⟨00*l*⟩ direction are characteristics of freestanding (*O*–*T*) superstructures which are grown seamlessly along the *c*-direction. In addition to the regular (00*l*) peaks with periodicities of 11.3 Å, the XRD pattern exhibits weaker reflections with periodicities of 17.2 Å, typical for the (*O*–*T*–*T*) superstructure.^37^ Indeed the purely (*O*–*T*–*T*) order in a nanotube/flake generates a new compound, i.e., (SmS)_1.19_(TaS_2_)_2_. Here, the (*O*–*T*–*T*) order was interspersed, sporadically, between the (*O*–*T*) superstructure and was probably caused by the defects in the SmS unit. The relative intensity of the (002) planes of (*O*–*T*) and (*O*–*T*–*T*) structures in XRD yields 4% of (*O*–*T*–*T*) layers in the overall compound. It is worth mentioning that a systematic transformation from (*O*–*T*), i.e., (LaS)_1.14_(TaS_*x*_Se_1–*x*_)_2_ to (*O*–*T*–*T*) (LaS)_1.14_(TaSe_2_)_2_ MLC, occurred upon increasing the selenium to sulfur ratio in the asymmetric misfit system.^37^ Further, the X-ray diffraction data (Figure S2) did not reveal any characteristic peaks for impurities, such as binary sulfides (SmS/TaS_2_) and elemental Sm/Ta/S in the reaction products within its sensitivity limit.

**Figure 2:**
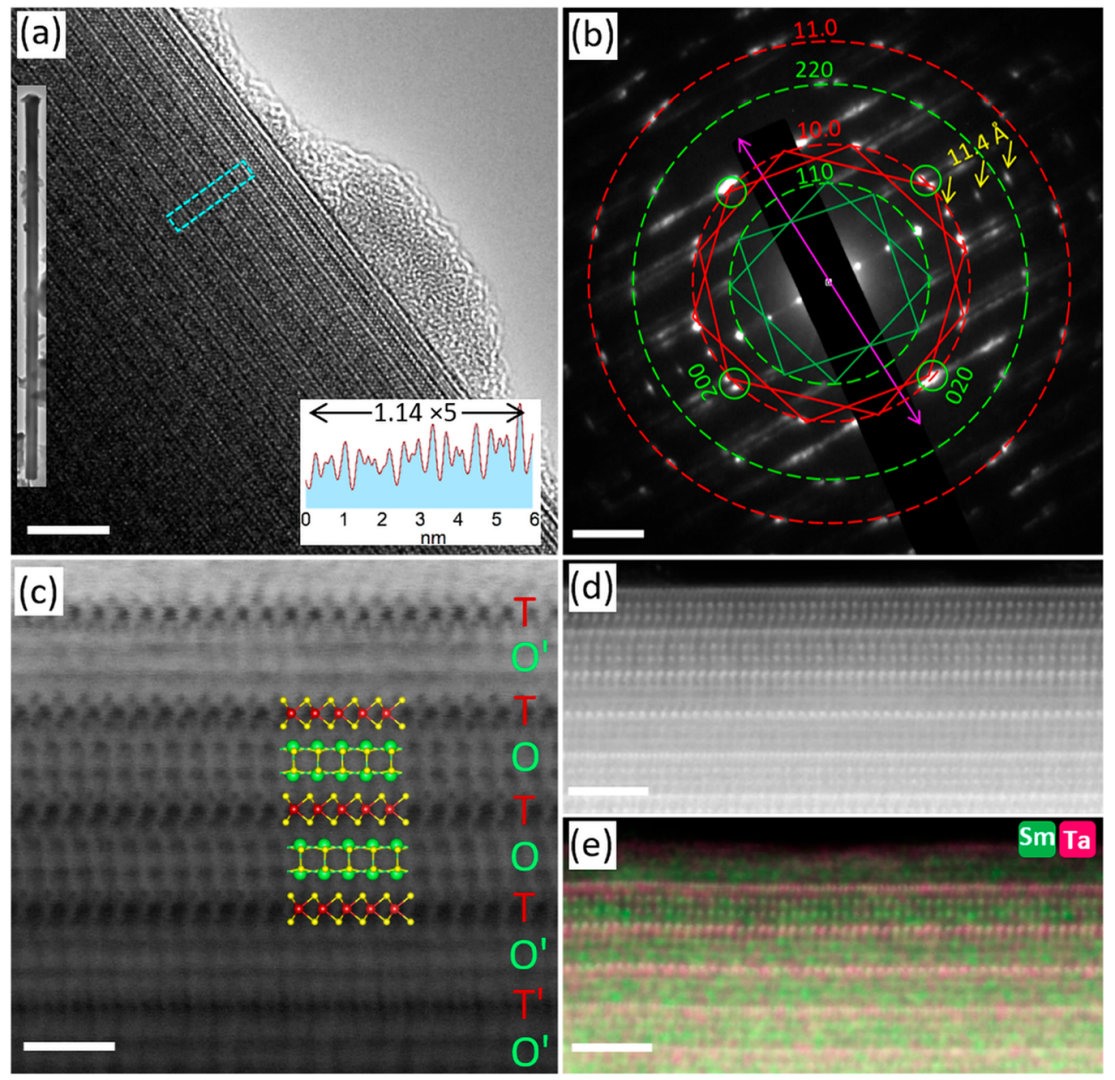
TEM images, electron diffraction, and chemical analyses of a (SmS)_1.19_TaS_2_ nanotube. (a) High-resolution TEM image. The periodic stacking of SmS and TaS_2_ layers in the misfit structure is revealed with TaS_2_ as the outermost layer. Scale bar is 5 nm. Low magnification image of the nanotube (diameter 170 nm) and an intensity line profile perpendicular to the tube axis are shown in the insets. (b) SAED pattern acquired from part of an individual nanotube. The sets of diffraction spots corresponding to SmS and TaS_2_ are marked with green and red dotted circles. The respective Miller indices are indicated. Small yellow arrows indicate basal plane reflections, and the tubule axis is marked by purple double-headed arrow. The two sets of four pairs of (110) reflections of the rocksalt SmS subsystem are marked by rotated green squares. The two sets of six pairs of (10.0) reflections of the orthohexagonal TaS_2_ sublattice are marked by red hexagons. These sets of reflections are rotated by 30° with respect to each other. (c) Atomically resolved HR-STEM image of a few (*O*–*T*) layers near the surface of the nanotube. The scale bar is 1 nm. The corresponding atomic model is overlaid on the HR-STEM image. The 30° rotation of (*O*–*T*)(*O*–*T*)′ layers is clearly visible. Yellow, red, and green spheres represent S, Ta, and Sm atoms, respectively. (d) STEM-HAADF image and overlaid (e) STEM-EDS elemental maps of Sm (red) and Ta (cyan) of a few layers from the surface of the tube; scale bar is 2 nm.

Transmission electron microscopy (TEM) analysis reported in previous studies^27^ did not have sufficient resolution to reveal the finest details of the structure of these nanotubes. Therefore, in the present work, the nanotubes were analyzed via TEM techniques with the highest possible resolution. Several (SmS)_1.19_TaS_2_ nanotubes were examined here. The results for one such nanotube are displayed in Figure 2 (see also Figures S3 and S4). A low magnification image of the nanotube in the inset of Figure 2a shows a constant diameter of 170 nm along its entire length. The high-resolution TEM image of the SmS-TaS_2_ superstructure reveals a periodic stacking sequence of SmS and TaS_2_ along the *c*-direction. The outermost layer is TaS_2_, which is true for all the nanotubes analyzed here as well as other Ln-based misfit nanotubes. Note that potential sources of damage prior to investigation, such as plasma cleaning, were avoided to preserve the pristine surface of the nanotubes. Previously, an STM study of the cleaved surface of SmS-TaS_2_ shows that SmS is the surface layer,^36^ but that observation may have resulted from the cleavage process.^36^ The surfaces of the nanotubes analyzed here are almost intact and, unlike LaS-TaS_2_,^51^ do not show any, or little, oxidation. The intensity profile drawn perpendicular to the tube axis (along *c*) shown in the inset reveals that the periodicity of the single-layer MLC unit is 11.4 Å, which is in close agreement with the (00*l*) reflection of the corresponding (*O*–*T*) structure observed from XRD.

The selected area electron diffraction (SAED) pattern of an individual (SmS)_1.19_TaS_2_ nanotube is displayed in Figure 2b. The intense and distinguished spots indicate ordered stacking of the SmS and TaS_2_ layers in the misfit lattice. The ED pattern reveals a pair of 4-fold and a pair of 6-fold periodicities for rocksalt SmS and hexagonal TaS_2_ sublattices, respectively, which are rotated by 30° with respect to each other. Two sets of four pairs of spots that are azimuthally equally distributed with the interplanar spacings of 1.85 and 3.7 Å (on the green circle) were assigned to the (110) and (220) planes of rocksalt SmS. Two sets of six pairs of spots (on the red circles) with the interplanar spacings 1.6 and 2.85 Å are attributed to hexagonal TaS_2_. These azimuthally equally distributed sets of quartet and sextet spots (marked by green squares and red hexagons, rotated by 30°) indicate two folding vectors for both SmS and TaS_2_ layers in the nanotube. This results in the formation of super periodicity of the kind (*O*–*T*)(*O*–*T*)′ and is reminiscent of the *CF* and *FF* superstructure in MLC (*vide infra*).^52^ The angular splitting of the spots into pairs is due to a small chiral angle of the nanotube (≈3°). The (020) reflections of SmS, which coincide with the (10.0) reflections of TaS_2_ (marked by a small green circle), reveal the common commensurate *b*-axis. The (020) and (200) reflections of SmS are approximately placed on the same dotted circle and are perpendicular to each other. This observation confirms that the *a* and *b* lattice parameters of SmS are (almost) equal. In the present nanotube, the common commensurate *b* axis appears 15° off from the nanotube growth axis. In many other nanotubes, the commensurate *b* axis coincides with the tube axis (see Figure S3). The basal plane reflections indicated by yellow arrows reveal the periodicity of 11.4 Å along the *c*-direction. In general, the (SmS)_1.19_TaS_2_ nanotubes prepared in this study exhibit a high degree of crystallinity, and the superstructure of SmS and TaS_2_ is well preserved.

The atomic structure of the nanotubes was analyzed with HR-STEM and STEM–EDS (Figure 2c–e and Figure S4). The atomically resolved STEM bright-field (BF) image in Figure 2c reveals that the nanotubes are comprised of (*O*–*T*) and (*O*–*T*)′ layers with high stacking order. The contrast difference between (*O*–*T*) and (*O*–*T*)′ layers is clearly visible, indicating the two different crystallographic orientations of the misfit layers. The interatomic distances in the projection reveal different orientations of the TaS_2_ in the nanotube; the viewing directions are along ⟨10.0⟩ and ⟨11.0⟩. Similarly, the orientation of the SmS layer can be linked to the ⟨100⟩ and ⟨110⟩ directions, which are in line with the ED results. In many nanotubes of this kind, the (*O*–*T*) pairs seem to be tilted 30° with respect to the adjacent (*O*–*T*)′ wall forming thereby a superperiodicity of the kind (*O*–*T*)(*O*–*T*)′ as confirmed by ED. The reason for the tilting of (*O*–*T*) layers is unknown, though. A possible explanation is that, quenching from the high temperature to room temperature in the synthesis may have arrested the reorganization of layers in the nanotube. Alternatively, the misfit lattice may prefer this unique orientation to minimize the misfit strain. The two different orientations in the nanotube tend to alternate along the common *c*-axis leading to double periodicity with 23 Å as the unit distance of the supercell (Figure 2c,d). With the first two layers being (*O*–*T*), i.e., ⟨10.0⟩ TaS_2_ and ⟨100⟩ SmS, the adjacent layers are oriented 30°, designated as (*O*–*T*)′, i.e., ⟨11.0⟩ TaS_2_ and ⟨110⟩ SmS, respectively. There is stronger contrast of the (*O*–*T*) layers over the (*O*–*T*)′ layers. The difference in contrast is related to orientation-dependent channeling phenomena and not to the composition. Careful analysis of the atomic arrangement reveals that the TaS_2_ lattice consists of a chevron-type pattern where the sulfur atoms are coordinated with the Ta atoms in trigonal prismatic fashion, i.e., the 1H polytype. Generally, quenching of TaS_2_ from such a high temperature would lead to the 1T polytype.^53^ The stability of 1H-TaS_2_ in the (SmS)_1.19_TaS_2_ MLC can be attributed to strong charge transfer from the SmS to TaS_2_, which is further confirmed by spectroscopic techniques (*vide infra*). The SmS subunit is arranged in a distorted rocksalt structure. In the ⟨100⟩ viewing direction of the SmS, the sulfur atoms sit at a small projected distance from the samarium, and consequently in this orientation sulfur and samarium positions are not as well resolved as below (see Figure 4b). The representative atomic models of ⟨10.0⟩ TaS_2_ and ⟨100⟩ SmS are overlaid on the HR-STEM image for clarity. The HR-STEM (DF) image and the corresponding HR-STEM-EDS maps overlaid on the STEM image are displayed in Figure 2d,e. The STEM-EDS confirms that Sm and Ta are in antiphase relationship with each other. Since the sulfur atoms are coordinated to both samarium and tantalum atoms, the sulfur maps show a uniform distribution due to channeling phenomena in the vicinity of the heavier Ta and Sm atomic helices and do not yield any extra information here.

**Figure 3:**
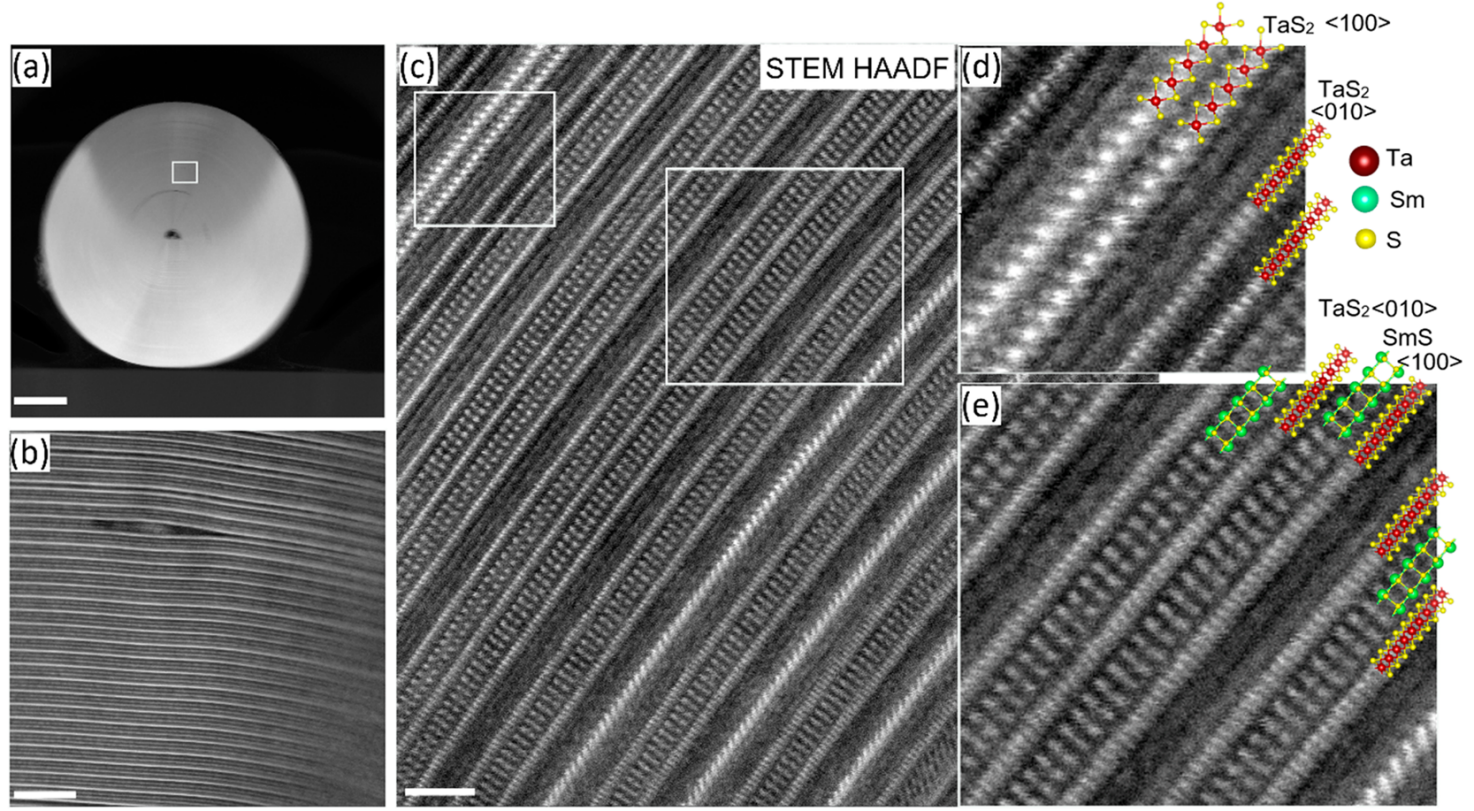
STEM analysis of cross-sectioned FIB lamella of a (SmS)_1.19_TaS_2_ nanotube. (a) Low magnification STEM-BF image of nanotube lamella; scale bar is 100 nm. (b) High magnification STEM-HAADF image of a portion of the cross-section showing the basic MLC backbone (*O*–*T*) structure; the defect layer of SmS ending abruptly is seen, and the scale bar is 5 nm. (c) Atomic resolution STEM-HAADF image revealing the rocksalt SmS and trigonal prismatic TaS_2_ layers; the rotation between the layers (*O*–*T*) and (*O*–*T*)′ is evident, and the scale bar is 2 nm. The SmS and TaS_2_ layers that are highly resolved correspond to (*O*–*T*) orientation, and those that are hardly resolved correspond to (*O*–*T*)′ orientation. (d and e) Atomic-resolution images from the area marked in image (c); projections of atomic models of double hexagonal TaS_2_ layers and distorted rocksalt SmS are overlaid on the STEM image.

To understand the structural details further, nanotubes were sliced into thin lamella using FIB microscope and transferred onto a TEM grid. Figure 3a shows a low-magnification STEM-ADF image of one such cross-section (the tube diameter is around 500 nm). A magnified image in Figure 3b shows an edge dislocation and, adjacent to it, a misfit structure with double layer periodicity (*O*–*T*–*T*). It is believed that the two features occur in the vicinity to one other due to strain fields induced by the dislocation. Generally, the confined volume and curvature of the nanotubes confer larger density of defects than the flakes.^37^ Moreover, the fact that each layer in the MLC nanotube contains a different number of atoms induces strain, which can be relaxed via defect formation. Figure 3c–e shows high-magnification STEM-BF images of a portion of the lamella. Indeed, the top exploded area includes three such repeating (*O*–*T*–*T*) units (Figure 3d). The repeating two TaS_2_ layers appear in trigonal prismatic coordination, i.e., the 2H polytype arrangement. The rotation of 30° in the (*O*–*T*–*T*) arrangement is also evident, i.e., TaS_2_ ⟨10.0⟩/SmS ⟨100⟩ and TaS_2_ ⟨01.0⟩/SmS ⟨110⟩. The fogging of some of the MLC layers in the HRSTEM images can be ascribed to the stacking faults, i.e., rotation of the (*O*–*T*) pair with respect to the (*O*–*T*)′ or (*O*–*T*–*T*) and (*O*–*T*–*T*)′ layers. The different crystallographic orientations of the two pairs leads to a small scattering of the incoming electron beam and blurring of the TEM image. On the other hand, the ordered structure from other portions of the lamellae consists of purely (*O*–*T*) superstructures (Figures 3e and S5), but the orientation of the layers varies from one pair to another. This observation reinforces the proposition that the (*O*–*T*–*T*) superstructure in this lamella indeed resulted from the defects formed by the SmS layer. Schematic rendering of the SmS ⟨110⟩ and TaS_2_ ⟨01.0⟩ atomic models corresponds very well to the underlying STEM-BF image.

**Figure 4:**
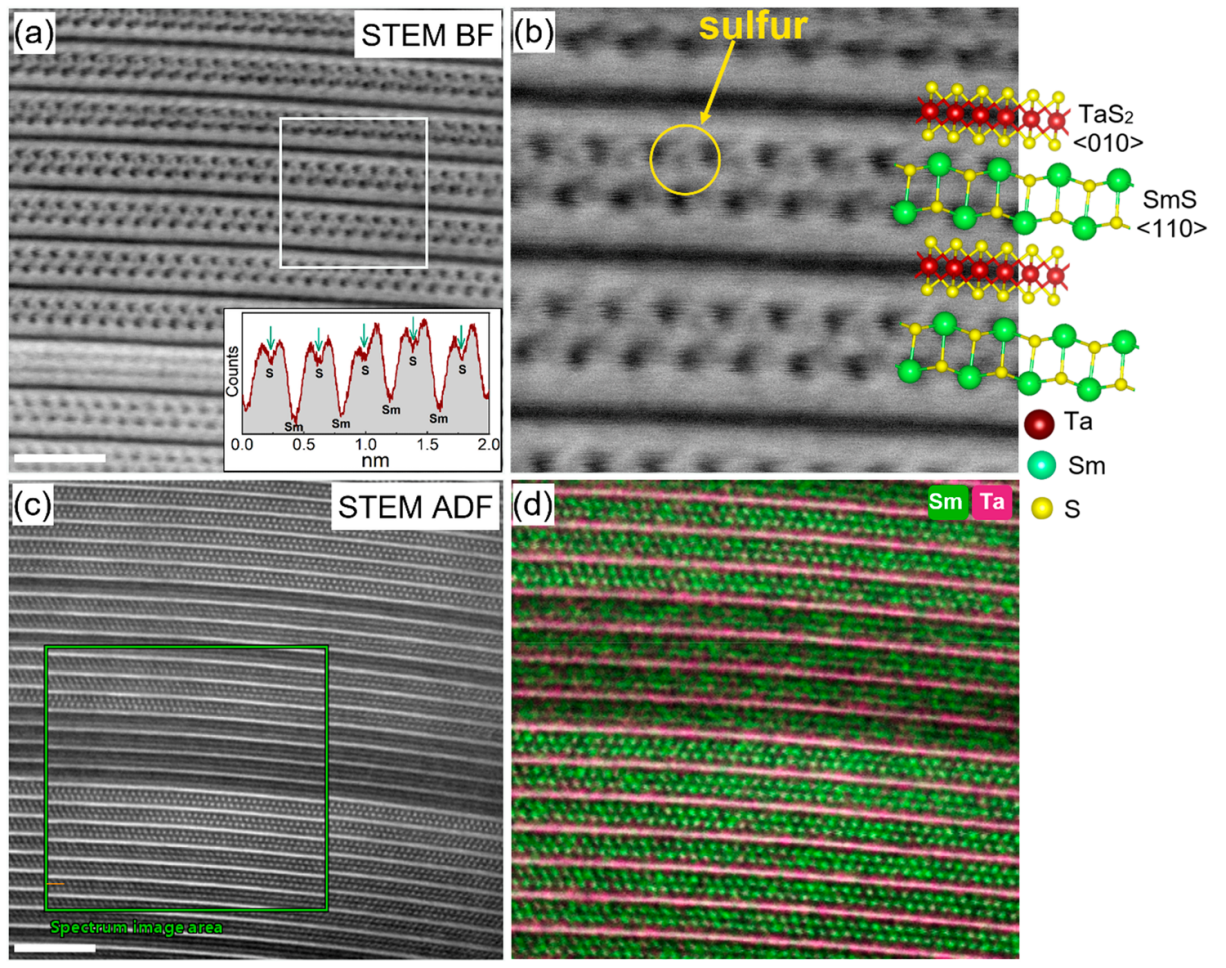
(a) Atomic resolution HR-STEM-BF image of a portion of the nanotube lamellae; a magnified image in (b) succinctly reveals the sulfur atoms adjacent to samarium atoms in the rock salt unit. The intensity profile is drawn from one of the SmS layers shown in the inset of (a), and it shows clear modulation of S and Sm; scale bar is 2 nm. (c and d) STEM-ADF and corresponding STEM-EDS analyses of (SmS)_1.19_TaS_2_ nanotube lamellae. EDS mapping shows the clear antiphase correlation between Sm and Ta atoms; scale bar is 5 nm. Sulfur atoms were distributed uniformly across the lamellae and are presented in the SI, Figure S6.

**Figure 5:**
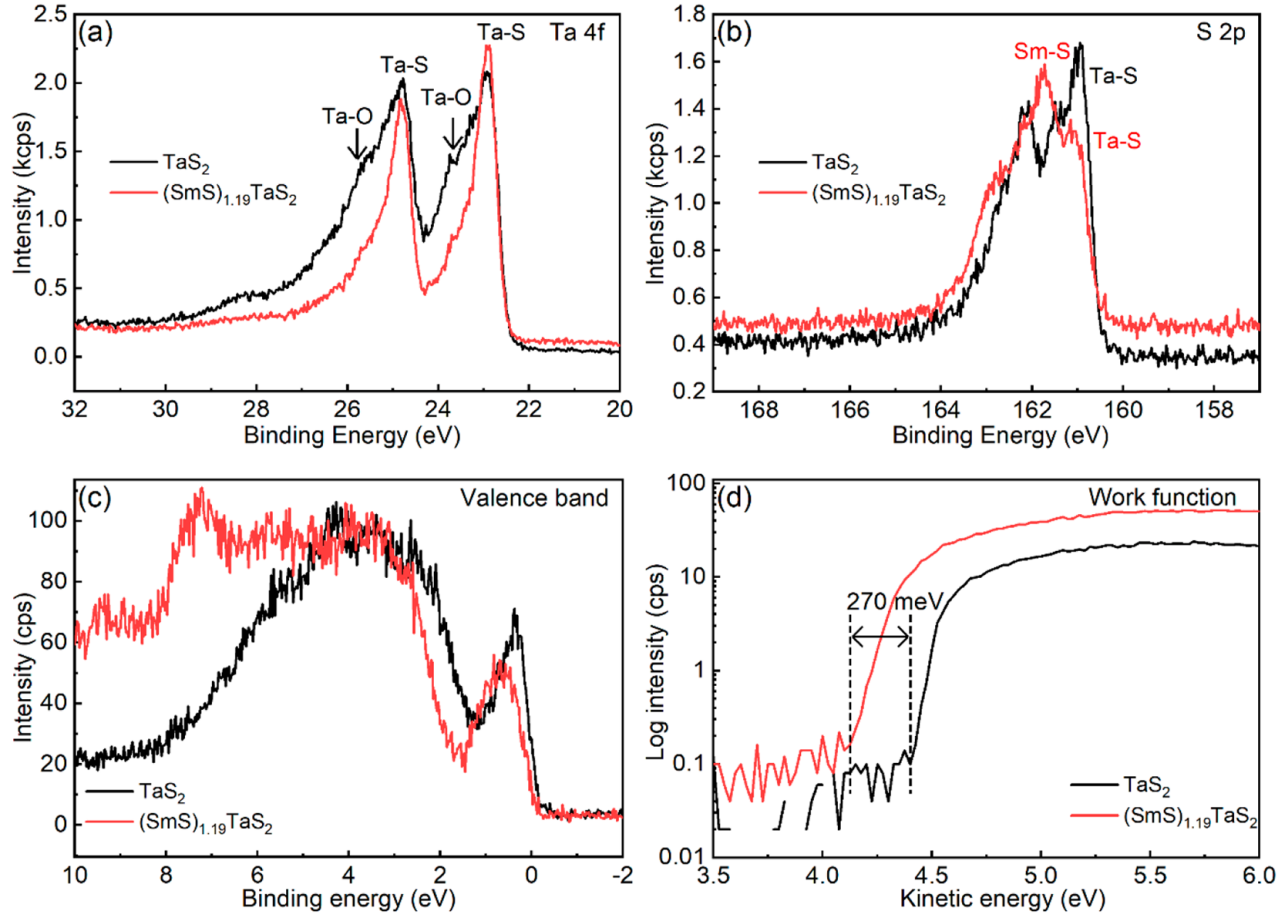
X-ray photoelectron spectroscopic results of TaS_2_ platelets (black) and the (SmS)_1.19_TaS_2_ misfit nanotubes (red). (a) The Ta 4*f* doublet; (b) the S 2*p* doublet; (c) the valence band spectral region; and (d) the onset of secondary electron emission, given on a log-scale, from which the sample work function is extracted for (SmS)_1.19_TaS_2_ and TaS_2_, respectively.

Figure 4a,b and Figure S5 show HR-STEM-BF images and exploded views of a lamella from a different region of the same nanotube. The (*O*–*T*) superstructure order is strictly followed, extended over many layers. Since the SmS layer is oriented along ⟨110⟩, the Sm and S atoms in the rocksalt structure are skewed and are therefore clearly visible in a magnified STEM-BF image (Figure 4b). The intensity profile drawn from one of the SmS layers (inset of Figure 4a) reveals clear modulation of the S and Sm atoms. Note that, since the rocksalt structure is distorted, the samarium and sulfur atoms do not share a common plane, i.e., the sulfur atoms are displaced toward the center of the SmS subunit. The high-resolution STEM-EDS images (Figures 4d and S6) show that the two Sm and Ta layers are in antiphase relationship with each other. The even sulfur distribution on the entire lattice is evident (see Figure S6a). The intensity profiles of the EDS chemical maps presented in Figure S6b show two Sm atomic layers (of the rocksalt unit) in between the Ta layers and their periodic modulation. The EDS sulfur profile reveals two atomic layers of trigonal prismatic coordinated sulfur adjacent to the Ta atomic layer, and the sulfur from the SmS layers is also distinguishable from that of the TaS_2_ layer. No indication for any oxide formation in the core of the nanotube was observed. The HR-STEM and STEM-EDS results presented here revealed atomically each Sm, Ta, and S atom and their positions in the MLC lattice as well as their distribution, which was not available before. The Ta–S atoms are in trigonal prismatic coordination in the hexagonal lattice while the Sm–S atoms are coordinated in a distorted rocksalt lattice with orthorhombic symmetry. Equipped with these insights, the stability of the misfit lattice gained upon charge transfer from the SmS slab to TaS_2_ was studied by combined XPS, EELS, and XAS analyses. These analyses were further corroborated by theoretical calculations, thereby shedding light on the structure–property relationships.

(SmS)_1.19_TaS_2_ powder containing nanotubes and flakes was densely spread over a carbon tape, and XPS spectra were collected from the sample surface. Figure 5 presents XPS measurements of the (SmS)_1.19_TaS_2_ misfit samples in comparison with 2H-TaS_2_ flakes. The core-level Ta 4*f* spectrum of pure TaS_2_, Figure 5a, shows pronounced oxidation as evident from the high energy shoulders at ∼24 and 26 eV of the Ta 4*f* doublet.^54^ This signal is believed to arise from the platelet edges that point upward in the powder grains. Curve fitting details of the Ta 4*f* doublet in TaS_2_ and (SmS)_1.19_TaS_2_ are presented in Figure S7. Remarkably, the Ta line appears far more homogeneous in the misfit sample, which indicates that nanotubes suffer much less edge oxidation than the (TaS_2_) platelets. The sulfur S 2*p* core-level spectra of TaS_2_ and the (SmS)_1.19_TaS_2_ misfit are presented in Figure 5b. Coexistence of the two misfit constituents is manifested by the S 2*p* spectrum (see also Figure S8). Here, as expected, two leading sulfur chemical states are observed, attributed to S in the trigonal prismatic TaS_2_ and the rocksalt SmS ingredients. For the reference TaS_2_ sample, the SmS component is missing from the S 2*p* line, while other components arise due to platelet edge oxidation (see also the Ta 4*f* line). The 3*d* core levels of samarium, observed at 1084 (Sm 3*d*_5/2_) and 1110 eV (Sm 3*d*_3/2_), are consistent with literature reports of the Sm^3+^ chemical state (see Figure S9).^55^ No signatures of Sm^2+^ at energies 1073 and 1100 eV were observed.^55^ Earlier reports of single-crystal (SmS)_1.19_TaS_2_ suggested Sm^2+^–Sm^3+^ valence fluctuation, but no evidence of that notion is seen here. Further support for this conjecture, i.e., the existence of pure Sm^3+^ and the absence of valence fluctuations, was provided by the XAS analysis (*vide infra*). Quantitative analysis of the chemical composition showed a Sm/Ta ratio of 1.12, which is slightly lower than the theoretical value, 1.19 and close to the values reported by SEM-EDS and STEM-EDS.

The XPS valence band spectra presented in Figure 5c show a clear difference between the reference (TaS_2_) and the (SmS)_1.19_TaS_2_ structure. The missing feature at 4–8 eV is attributed to the lack of Sm contribution. Differences at the top of the valence band, just below the Fermi energy (zero binding energy), are seen as well. The valence band spectrum of (SmS)_1.19_TaS_2_ exhibits broadening plus a shift of the MLC spectrum to higher binding energies compared to the pure TaS_2_. This result suggests that the Fermi level of the misfit was “pushed” upward and the misfit became less *p*-type, as compared to TaS_2_. These differences are complemented by the work function (WF) shift shown in Figure 5d. Here, a clear difference in the onset of the secondary electron emission, about 270 meV in magnitude, is seen between the reference and the MLC. The latter result suggests that the WF of bulk SmS is lower than that of TaS_2_, hence when brought into contact with TaS_2_ (in the MLC), electron density is expected to be transferred from the SmS to the TaS_2_ layers. Consequently, the valence band of the semiconducting SmS is partially depopulated (by charge transfer) and thus becomes conductive as well. Interestingly, the Ta 4f_7/2_ binding energy of the TaS_2_-related component in the misfit compound is similar to the one in pure TaS_2_. This fact indicates that the donated charge contributes a charge density to the Ta atoms exclusively, such that both the Fermi energy and the Ta-core levels are equally affected (see also the SI).

Notably, XPS measurements can also provide rich information on the electrical properties of the probed samples.^56−59^ In the present study, tested under extreme positive and negative charging conditions, both TaS_2_ and SmS-TaS_2_ are found to be very good conductors. Advantageously, these measurements, done *in-situ* in the XPS chamber, offer contactless electrical characterization. Yet, as an electrical probe, these measurements are better suited for semiconductors and insulators, yielding only limited sensitivity to the differences between the conductivity levels of metals. Notwithstanding this reservation, the conductivity of the misfit sample was found to be very similar to that of the metallic TaS_2_ platelets. Hence, this observation suggests, in agreement with previous reports,^7^ that band filling by the Sm-to-Ta charge transfer is incomplete and the metallic conductivity is preserved in both constituents of the MLC.

**Figure 6:**
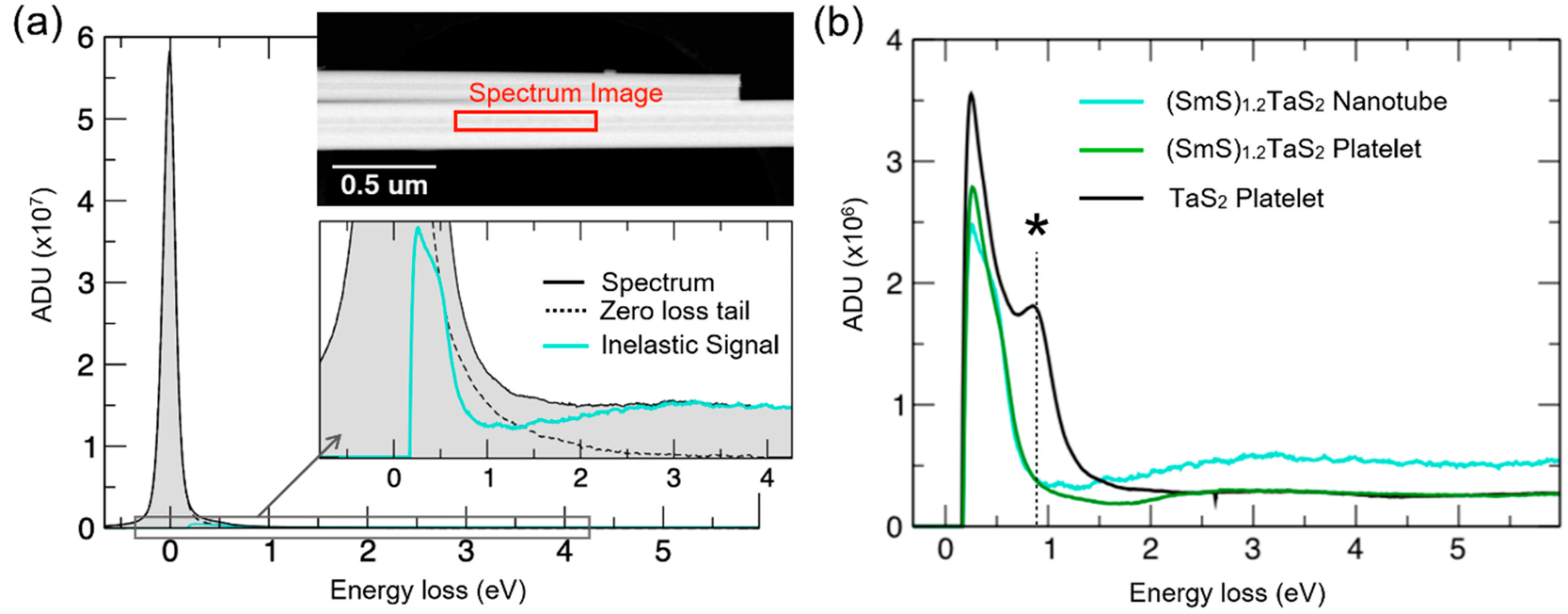
Low-loss EEL spectra. (a) An exemplary spectrum of a (SmS)_1.19_TaS_2_ nanotube. The insets show an annular dark-field image of the nanotube and a magnified part of the spectrum with the extracted inelastic signal. The spectrum is a sum of spectra obtained in a region of interest (red rectangle) close to the tube axis, where the incident direction of the electron beam is close to the *c*-axis direction of the MLC. For background subtraction, the tail of the elastic zero-loss peak was reflected from the high-energy side. (b) Inelastic part of the EEL low loss signal for three samples, the center part of a (SmS)_1.19_TaS_2_ nanotube (as in (a)), a (SmS)_1.19_TaS_2_ platelet, and 2H-TaS_2_ platelets. The platelets were transmitted in the direction of the *c*-axis. The strong peak at about 1 eV energy loss (marked by an asterisk) in 2H-TaS_2_ is associated with a transition from occupied S 3*p* states into unoccupied Ta 5*d*_*z*^2^_ states. The peak is absent in the MLC, in the platelet, and in the nanotube.

Monochromated low-loss EEL spectra of TaS_2_ and the (SmS)_1.19_TaS_2_ misfit compound are presented in Figure 6. Hyperspectral data were recorded with a scanning focused probe obtaining both spatial and a high energy resolution (better than 90 meV) to resolve low-energy excitations in the near-infrared region. Figure 6a displays an exemplary spectrum of a (SmS)_1.19_TaS_2_ nanotube, obtained as the sum of multiple spectra in a region of interest across the central part of the nanotube. The elastic contribution to the spectrum was subtracted using the mirrored left-hand tail of the zero-loss-peak (ZLP).^60^ The remaining tail at the lowest energies in the inelastic part of the spectrum contains Cerenkov losses and surface losses that are flattened out here because of a large collection angle. The inelastic part of the low loss spectrum is plotted for the central part of a (SmS)_1.19_TaS_2_ nanotube, the edge of a (SmS)_1.2_TaS_2_ platelet, and a 2H-TaS_2_ platelet in Figure 6b. The reference spectrum of 2H-TaS_2_ is very distinct from those of the misfit compound: A strong transition at around 1 eV observable all across the platelets of TaS_2_ is almost absent in the misfit layered compound. To understand the origin of this peak and the reason for its absence in the misfit compound one has to refer to the classical dielectric formalism of the loss function.^61,62^ Accordingly, the EEL low-loss function is related to the so-called volume loss function, i.e., Im(−1/ε), where ε is the frequency-dependent dielectric function. The imaginary (ε_2_) and real (ε_1_) parts of the frequency-dependent dielectric functions for bulk 2H-TaS_2_, obtained from DFT calculations, are presented in Figure S10. A peak in ε_2_ of 2H-TaS_2_ just above 1 eV upon longitudinal excitation along the *c*-axis can be possibly ascribed to a transition from occupied S 3*p* states into unoccupied Ta 5*d*_*z*^2^_ states above the Fermi level. Such an S 3*p* to Ta 5*d* transition is indeed likely to apply for the ∼1 eV loss in the TaS_2_ EEL spectrum, because it does not violate the dipole selection rules. Yet, the intensity of this peak is particularly high, and therefore, the related transition is suspected to be of a significant intra-atomic character, other than interatomic. This transition is enabled by intra-atomic transitions between, e.g., 5*d* Ta 3/2 and 5/2 states. Remarkably, the 1 eV EELS peak is practically absent from the (SmS)_1.19_TaS_2_ nanotubes and platelets. This result can be attributed to an almost complete filling of the Ta 5*d*_*z*^2^_ states in the misfit compound. The DFT calculations further substantiate an almost complete occupation of these Ta *d* states (*vide infra*). In agreement with the EEL data, the theoretical dielectric function of (SmS)_1.2_TaS_2_ obtained by DFT calculations does not show the excitation at about 1 eV (Figure S10) owing to the charge transfer from the Sm 4*f*-band to the Ta 5*d*_*z*^2^_ states in the misfit compound.

**Figure 7:**
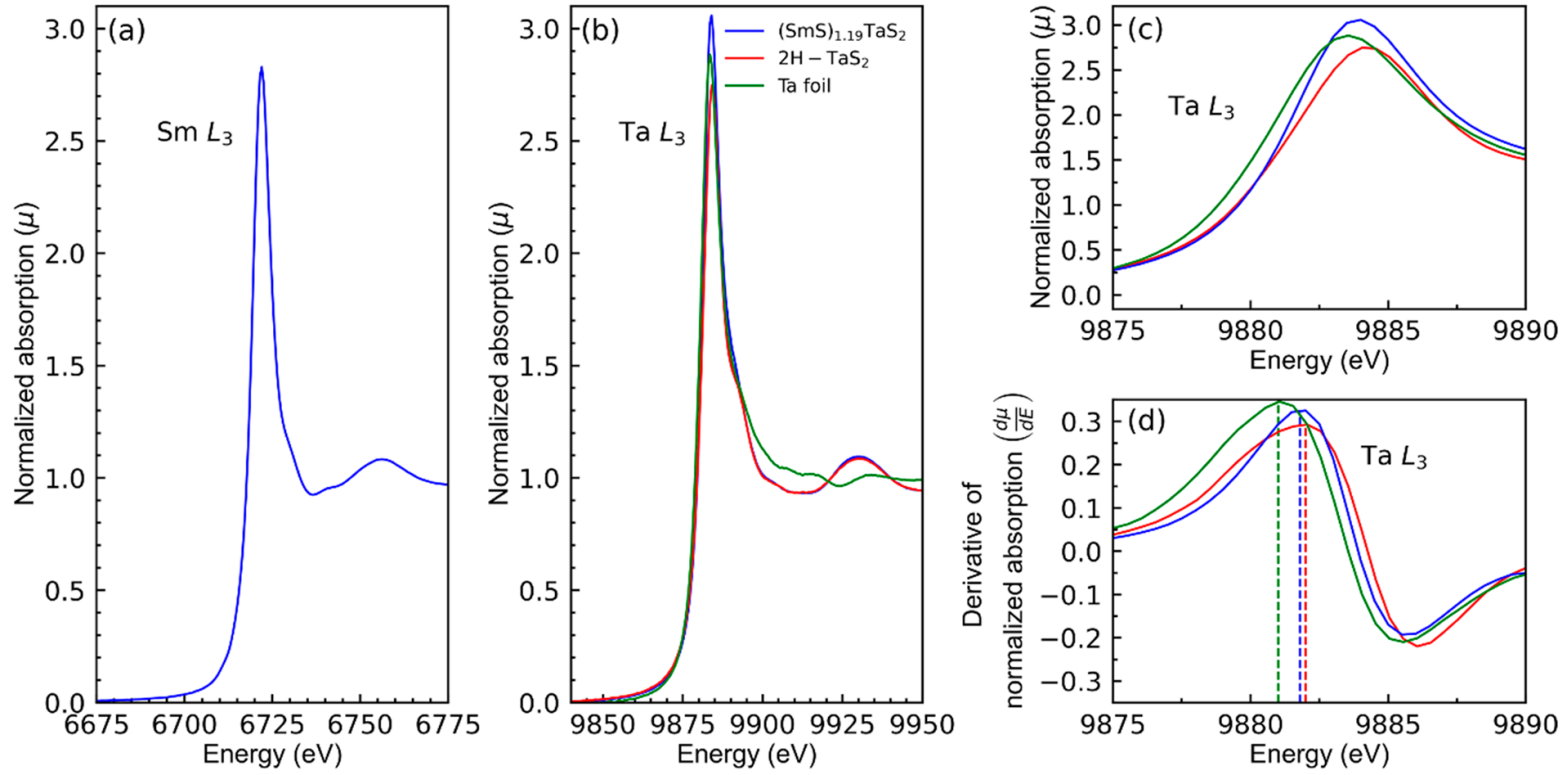
XANES spectra of (SmS)_1.19_TaS_2_ and TaS_2_ powders dispersed in the polymer matrix and pure (99.99%) Ta foil collected in a transmission geometry at PETRA III P23 beamline. Spectral regions for Sm-L_3_ (a) and Ta-L_3_ (b, c) edges. (d) Derivative of the normalized absorption in the vicinity of the Ta L_3_ edge. XANES in the (a), (b), and (c) spectra are normalized to the edge step, and the energy scale is calibrated with pure Ta and Mn foils.

Unlike the surface-sensitive XPS, XAS analysis provides the finest structural, chemical state, and charge transfer insights of the bulk. Figure 7 shows Sm L_3_ and Ta L_3_ XANES spectra of the (SmS)_1.19_TaS_2_ misfit compound (nanotube + flakes) in comparison with 2H-TaS_2_ and Ta foil (99.99%) collected in transmission geometry. The shape of the Sm L_3_ XANES spectrum (Figure 7a) and the position of the “white line” maxima at 6722 eV (edge position 6719.5 eV, corresponding to 2*p* to 5*d* transition) is characteristic of the Sm^3+^ state.^63^ No signature of the Sm^2+^ “white line” in the range 6711–6713 eV was found, indicating that there is no mixed/intermediate valence state of Sm, which in turn signifies a strong charge transfer.^63−65^ This observation is in-line with the XPS and also with the literature reports.^7,8^

The shape of the Ta L_3_ XANES spectrum (Figure 7b) resembles the 2H-TaS_2_ spectra, and the position of the “white line” at 9884 eV is close to that of the 2H-TaS_2_ structure.^66,67^ However, precise examination of the “white line” (Figure 7c) or the absorption edge position from the derivative of the normalized absorption (Figure 7d) shows that there is a small shift between 2H-TaS_2_ (9881.8 ± 0.1 eV) and the (SmS)_1.19_TaS_2_ (9882 ± 0.1 eV) Ta L_3_ edges. It is known that the position of the Ta L_3_ edge depends on the valence state of Ta: the higher the valence (oxidation state), the stronger the Ta L_3_ edge shifts toward higher X-ray energies.^68,69^Figure 7d shows that the position of the Ta L_3_ edge of pure Ta foil has a slightly lower value (oxidation state = 0, edge position 9881 ± 0.1) than those of TaS_2_ and (SmS)_1.19_TaS_2_. The small difference between the L_3_ edges of TaS_2_ and (SmS)_1.19_TaS_2_ (0.2 ± 0.1 eV) cannot be overinterpreted, since some tantalum oxide may have occurred on the surface of the tubes (flakes). The (SmS)_1.19_TaS_2_ Ta L_3_ “white” line intensity (3.01 ± 0.01), which corresponds to the transition from the 2*p* core level to the unoccupied Ta 5*d* states (*d*_*z*^2^_ band), is higher than the “white line” intensity value measured for pristine 2H-TaS_2_ (2.75 ± 0.01).^66,70^ Interestingly, the intercalation of pristine 2H-TaS_2_ by pyridine^66^ or hydrazine^70^ increases the intensity of the “white line” up to the values of 2.5 and 2.9, respectively. In the case of (SmS)_1.19_TaS_2_, the charge transfer from SmS to TaS_2_ would lead to a similar increase in the white line intensity of Ta L_3_ in comparison with pristine 2H-TaS_2_. In general, XAS investigations show that the white line intensity grows with electron-donating intercalates and diminishes with electron-withdrawing intercalates.^66,70^ The fundamental reason for such a change is yet to be understood.

### Density Functional Theory (DFT)

**Figure 8:**
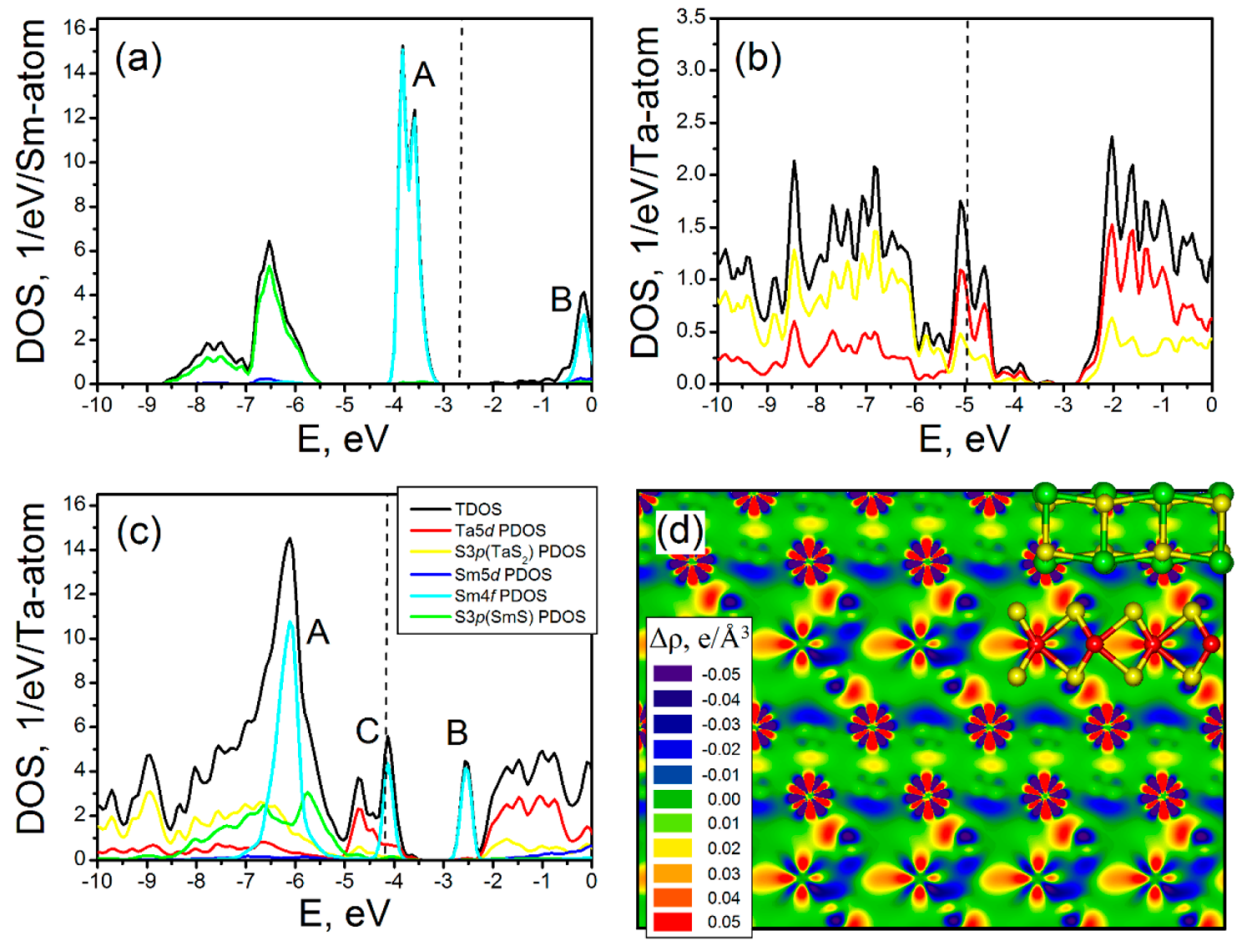
Electronic densities-of-states (DOSs) for (a) bulk fcc-SmS, (b) 2H-TaS_2_, and (c) misfit (SmS)_1.20_TaS_2_. The Fermi level is drawn as a dashed line. Panel (d) depicts the electronic density redistribution map after the (SmS)_1.20_TaS_2_ crystal assembly from SmS slabs and TaS_2_ monolayers. Sm, S, and Ta atoms of the ball-and-stick models are painted in green, yellow, and red, respectively. Corresponding spin-resolved band structures are plotted in Figure S11. DFT LDA+U calculations.

DFT calculations were employed to get an insight into the electronic structure of the SmS-TaS_2_ MLC and to compare it to the parent SmS and TaS_2_ phases. As a misfit model, the approximant (SmS)_1.20_TaS_2_ was chosen, in which a supercell included one SmS slab (12 SmS units) and one TaS_2_ layer (10 TaS_2_ units). A preliminary geometry optimization yielded the lattice parameters for the misfit as *a* = 17.08 Å, *b* = 5.79 Å, and *c*/2 = 11.53 Å. The in-plane parameters fitting for the TaS_2_ sublattice were close to that of bulk 2H-TaS_2_ (calc. *a* = 3.35 Å). On the other hand, the SmS sublattice showed a slight contraction compared to the bulk compound. Little peculiarity can be observed in the distortion of the SmS slab within the SmS-TaS_2_ misfit, when compared to the LaS slab within the LaS-TaS_2_ misfits studied earlier.^26^ The angles of the S–Sm–S configurations in this slab vary in the range 80–89° with the S atoms retracting into the slab, which is in agreement with the atom positions in the atomic-resolution STEM-BF images (see ball-and-stick model in Figure 8).

While the calculations within the local density approximation or generalized gradient approximation (LDA or GGA) ascribe a semimetallic character for bulk SmS, the present LDA+U calculations describe this compound as a semiconductor with a direct Γ–Γ transition type and a fundamental band gap of 0.69 eV (Figure 8a and Figure S11). The latter is consistent with the scattering in the available experimental and theoretical data. The experimentally reported gap is 0.15 eV^71^ or 0.4 eV,^72^ whereas the calculated values are 0.25 eV^72^ or 0.71 eV.^73^ The band gap edges arise from the highly intense and strongly localized band of occupied Sm 4*f* states and from the shallow band of unoccupied Sm 6*s* states. The valence band of the occupied S 3*p* states is found at 2.5–5.5 eV below the top of the Sm 4*f* band. In general, such a DOS profile characterizes SmS as a lattice with a highly ionic character. The nominal oxidation states of the elements are Sm^2+^ and S^2–^, and the Sm 4*f* states do not participate in the chemical bonding. Remarkably, the occupation and composition of the conduction band in the electronic structure of SmS are different from those of the isostructural LaS. In the latter, the Fermi level is hosted at a shallow band of La 5*d* states as shown in Figure S11.

According to the present and also earlier calculations, bulk 2H-TaS_2_ is a metal, where the Fermi level is hosted at the band of well-localized Ta 5*d*_*z*^2^_ states; see Figure 8b. The wide valence band is composed of a mixture of dominating S 3*p* and secondary Ta 5*d* states responsible for the covalent Ta–S bonding. The wide conduction band is dominated by the Ta 5*d* and secondary S 3*p* states and is separated from the Ta 5*d*_*z*^2^_ band by a gap of ∼0.5 eV. The latter reflects the well-documented tendency of TaS_2_ to act as an acceptor within misfit compounds, (almost) filling the 5*d*_*z*^2^_ band similar to 4*d*_*z*^2^_ or 5*d*_*z*^2^_ bands in the semiconducting MoS_2_ or WS_2_.

In analogy with the (La,Y)S-TaS_2_ misfits studied in the past,^25^ (SmS)_1.19_TaS_2_ possesses a metal-like character in the framework of LDA+U calculations (Figure 8c). Here, the metallic properties of SmS-TaS_2_ arise due to the charge transfer from Sm 4*f* to Ta 5*d*_*z*^2^_ states within individual SmS and TaS_2_ components acting as the donor and the acceptor of the electron density, respectively. The absolute value of the Fermi level in SmS-TaS_2_ is found to be in between the Fermi levels of the parent binary compounds (Figure S11). The calculated effective charge on Sm atoms increases from +0.53 e in SmS to the average value of +0.81 e in (SmS)_1.20_TaS_2_, while the effective magnetic moments on all these atoms decrease from 6.97 μ_B_ to 6.49 μ_B_ on the average, respectively.

In contrast to (La, Y)S-TaS_2_ misfits, the DOS profile of the SmS-TaS_2_ misfit cannot be assembled from the DOS profiles for the individual SmS and TaS_2_ in a simplified rigid-band model. The charge transfer in SmS-TaS_2_ is accompanied by remarkable reorganization of the Sm 4*f* states compared to the pristine SmS compound (see bands A, B, and C in Figure 8a,c). The Fermi level is hosted at the shoulder of a Sm 4*f* band (C-band), which is split off from the main occupied Sm 4*f* band (A-band). This new C-band is also well localized and appears in the pristine SmS compound, nearly in the middle between occupied A-band and unoccupied B-band. Noteworthy, the A-band in SmS-TaS_2_ is aligned with both the occupied S 3*p* band of SmS and the occupied S 3*p* band of TaS_2_, which may point to additional strengthening of Sm–S bonding both within the SmS layer and at the SmS||TaS_2_ interface. Indeed, mapping the electron density redistribution within (SmS)_1.20_TaS_2_ unveils not only an enhancement of electron density at the Ta atoms (four-lobed patterns of Ta 5*d* orbitals in Figure 8d), but also a charge redistribution within the Sm atoms (six-lobed patterns of Sm 4*f* orbitals). Furthermore, an enhancement of electron density between the Sm atoms and the S atoms of TaS_2_ is visible, which is responsible for the rise of a new coordinate Sm–S bonding (red “blobs” between Sm and S). A slight enhancement of the electron density between Sm and S atoms within the SmS unit can be also observed.

To complement the data of low-loss EEL spectroscopy, the frequency-dependent dielectric functions ε(ω) = ε_1_(ω) + iε_2_(ω), where ε_1_(ω) and ε_2_(ω) are the real and imaginary parts of the function, respectively, have been calculated using the same pseudopotential DFT method for in-plane (xx) and out-of-plane (zz) scattering on both 2H-TaS_2_ and (SmS)_1.20_TaS_2_ compounds (Figure S10). The results for TaS_2_ are in semiqualitative agreement with the optical properties elucidated from the EELS analysis (Figure 6) and more sophisticated full-potential plane-wave calculations.^74^ The origin of the signal at ∼1 eV in the inelastic part of the low-loss EEL spectrum along the *c*-axis of TaS_2_ and the corresponding maximum of the ε_2_^zz^(ω) function at ∼1.5 eV in the calculations are related to the electron transfer into the half-filled Ta 5*d*_*z*^2^_ band; see Figure S10a. Contributions to these transitions arise from the S 3*p* and Ta 5*d* states (Figure 8b), noting that both of them are dipole-allowed (Δ*j* = ±1) transitions. The real part of the dielectric function, ε_1_, influences as well the 1 eV region. Yet, in common to all related transitions, the role of the empty Ta 5*d*_*z*^2^_ states is dominant. In contrast, for the misfit structure, the ∼1 eV region is modified significantly, due to the charge transfer discussed above. Accordingly, the dielectric function calculated for (SmS)_1.20_TaS_2_ confirms the disappearance of the EEL signal from the ∼1 eV regime, when applied along the *c*-axis of the MLC (Figure S10b).

### Magnetic Measurements

**Figure 9:**
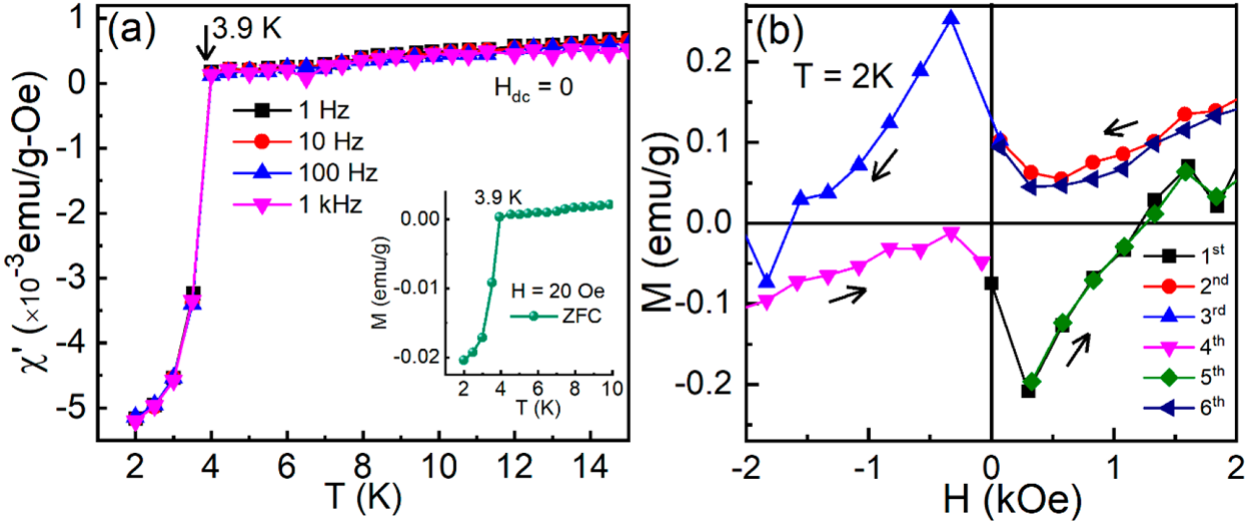
Temperature-dependent magnetic susceptibility of (SmS)_1.19_TaS_2_ MLC. (a) Real part of the ac magnetic susceptibility measured at frequencies *f* = 1, 10, 100, and 1000 Hz and *H*_dc_ = 0. Inset in (a) shows zero-field cooled dc magnetization as a function of temperature measured at *H*_dc_ = 20 Oe. (b) Zero field cooled isothermal magnetization measured at *T* = 2 K. The hysteresis loops were carried out as 0 → +10 (first cycle) → −10 (second and third cycles) → +10 (fourth and fifth cycles) → 0 kOe (sixth cycle). Only the part of hysteresis loops is shown for clarity.

The ac susceptibility measured at frequencies 1, 100, and 1000 Hz and zero dc magnetic field show a strong diamagnetic signal below 3.9 K (Figure 9a), indicating that (SmS)_1.19_TaS_2_ undergoes a superconducting transition below 5 K. This transition temperature is appreciably higher than that occurring in 2H-TaS_2_ (0.63 K).^52^ The presence of diamagnetic signals is also verified by zero-field cooled dc magnetization measured at a magnetic field of 20 Oe (Figure 9a, inset), and the transition temperature obtained by both ac and dc measurements is in excellent agreement. The low-temperature ac susceptibility shows a large volume fraction of ∼45%, of the superconducting phase, indicating the bulk superconducting in nature. The isothermal magnetization, *M*(*H*), measured at 2 K shows butterfly shaped hysteresis loops (Figure 9b) possibly associated with Type-II superconductivity.^75^ The observed behavior was verified three times for the (SmS)_1.19_TaS_2_ sample prepared in different batches. No superconductivity transition was observed in the magnetic measurements of the GdS-TaS_2_ tubes and flakes, which were prepared in a similar way (see Figure S12). The iso-field, iso-thermal magnetic, and ac susceptibility measurements show that GdS-TaS_2_ ordered antiferromagnetically at 7 K (Figure S12).

Charge density waves (CDWs) and superconductivity (SC) coexist in 2H-TaS_2_.^76^ It is well understood that the intercalation of alkali metals such as Li/Na and pyridine would enhance the superconducting transition temperatures and suppress the CDW transition.^76^ The intercalation of small quantities of Li or pyridine in 2H-TaS_2_ was shown to enhance the SC critical temperature, *T*_*c*_, from 0.8 to 3.5 K.^76^ A similar effect can be anticipated in misfit compounds, whereby the enhancement in *T*_*c*_ of (SmS)_1.19_TaS_2_ compared to pure TaS_2_ is reminiscent of the strong charge transfer from SmS to TaS_2_. The superconductivity in a series of MLCs was studied before using heat capacity and magnetic susceptibility analyses.^77^ The authors did not find any superconductivity in LaS-NbS_2_ and SmS-NbS_2_, which was attributed to the strong charge transfer between the layers and the stronger polar coupling between the layers, compared to other MLCs. Note that no evidence for the presence of pure tantalum impurities (*T*_*c*_ = 4.4 K) was obtained by any of the techniques used in this study. The magnetic susceptibility of SmS-TaS_2_ under relatively high magnetic fields (0.875 T) did not exhibit a superconductivity transition.^7^ The χ^–1^ vs temperature curve in the interval 100 K < *T* < 300 K shows good agreement (*R*-factor = 0.99987) with the Curie–Weiss equation (Figure S13); the derived Curie constant is *C* = 0.0627(6) cm^3^ K mol^–1^. The effective magnetic moment is equal to μ_eff_ = 0.71 μ_B_, while the calculated value of μ_eff_ for Sm^3+^ is 0.84 μ_B_.^78^ Thus, samarium in (SmS)_1.2_TaS_2_ MLC exists in the Sm^3+^ state with 4*f*^5^ (^6^H_5/2_) electronic configuration.

### Transport Measurements

**Figure 10:**
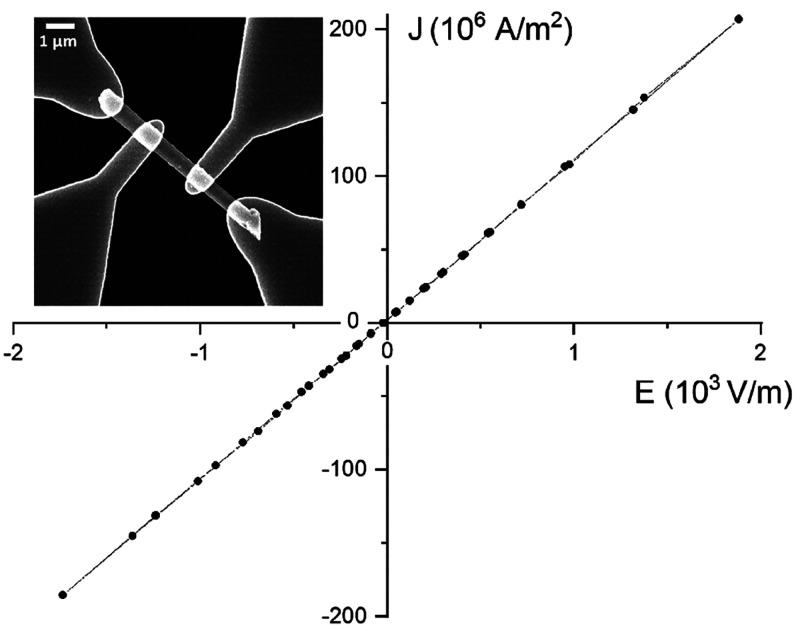
Typical *I*–*V* curve of a single (SmS)_1.19_TaS_2_ nanotube (550 nm in diameter and 7 μm long), giving resistivity ρ = 0.92 × 10^–3^ Ω·cm. Several devices of this kind were prepared, exhibiting resistivities between 0.40 × 10^–3^ Ω·cm and 0.95 × 10^–3^ Ω·cm. The inset is an SEM image of a nanotube (in the middle) with four contact probes fabricated using EBL, showing the geometry of devices used for (room temperature) electrical transport measurements.

Transport properties of individual LaS-TaS_2_ nanotubes were recently reported.^51^ In support of the DFT calculations, the LaS-TaS_2_ nanotubes were found to be semimetallic. Given the fact that the Sm-based MLC is chemically more stable than the LaS-TaS_2_ and the improved handling of the nanotubes, surface oxidation of the nanotubes were generally less of an issue here. Several devices of this kind were prepared and measured (Figure 10). The specific resistivities derived from the measurements are in the range of (0.40–0.95) × 10^–3^Ω·cm. These resistivity values are similar to the room temperature resistivity of bulk 2H-TaS_2_, which is on the order of 5 × 10^–3^ Ω·cm.^79^ In fact, these values are comparable to the values reported in ref (7). The somewhat counterintuitive low resistivity of SmS-TaS_2_ nanotubes, which is comparable to that of 2H-TaS_2_ flakes, can be possibly attributed to their quasi-1D structure, which leads to a reduced scattering of the charges. Preliminary cathodoluminescence (CL) measurements of individual nanotubes in cryogenic temperatures (−150 K) were carried-out within the SEM. The CL spectra revealed *f*–*f* luminescence with a peak at 695 nm (see Figure S14), which was absent in the background. The 695 nm luminescence peak has been previously assigned to Sm^2+^ and is attributed to the local reduction of Sm^3+^ by the electron beam.^80^

## Conclusions

Herein, the structure and some physical properties of nanotubes and flakes formed from the misfit layered compound (SmS)_1.2_TaS_2_ were studied. High-resolution transmission electron microscopy was used to shed light on the structure of the nanotubes in detail. Their cross-sectional lamella were prepared and studied via HR-STEM providing unprecedented resolution of the lattice atoms in the nanotube. In particular, the trigonal prismatic arrangement of the 2H-TaS_2_ was clearly visible. X-ray photoelectron spectroscopy and X-ray absorption and electron energy loss spectroscopy analyses coupled with density functional theory calculations indicated that strong charge transfer from the samarium 4*f* level to the 5*d*_*z*^2^_ levels in TaS_2_ leads to the partial filling of these energy levels. This charge transfer has several implications. First, the Sm atom is present as the Sm^3+^ valence state in the MLC lattice. Also, the SmS-TaS_2_ exhibits a comparable conductivity to bulk 2H-TaS_2_, which is vindicated through four probe transport measurements. Finally, the charge transfer suppresses the charge density wave phase of TaS_2_, promoting thereby the superconductivity of this MLC with *T*_*c*_ of 3.8–4.4 K compared to 0.8 K for bulk 2H-TaS_2_. This study sheds new light on the structure–property relationships in MLC and their nanotubes in particular. In particular, the present study demonstrates that Ln-based MLC nanotubes are likely to exhibit intriguing 1D quantum physical phenomena at cryogenic temperatures.

## Supporting Information Available

Experimental and characterization details, computation details, XRD, HR-STEM, STEM-EDS, magnetic measurements, electrical characterization, frequency-dependent dielectric function, band structures of LaS-TaS_2_ and SmS-TaS_2_, magnetic susceptibility fitting of the χ(*T*) Curie–Weiss equation, and cathodoluminescence (PDF)

## Dedication

Dedicated to Professor John B. Goodenough on the Occasion of his 100th year Birthday. Until hundred and twenty like twenty—a proverb in Hebrew.
