# Supplementary material for: Nanotubes from the Misfit Layered Compound (SmS)1.19TaS2: Atomic Structure, Charge Transfer, and Electrical Properties
Source: Chem Mater. 2022 Feb 10;34(4):1838–53. doi: 10.1021/acs.chemmater.1c04106 (PMC8874355; doi:10.1021/acs.chemmater.1c04106)
Supplement: Supplementary file 1 — cm1c04106_si_001.pdf [file cm1c04106_si_001.pdf]

Supporting information for

Nanotubes from the misfit layered compound $(SmS)_{1.19}TaS_2$: Atomic structure, charge-transfer and electrical properties

M. B. Sreedhara,[1] Kristýna Bukvišová,[2] Azat Khadiev,[3] Daniel Citterberg,[2] Hagai Cohen,[4] Viktor Balema,[5,6] Arjun K. Pathak,[7] Dmitri Novikov,[3] Gregory Leitus,[4] Ifat Kaplan-Ashiri,[4] Miroslav Kolíbal,[2,8*] Andrey N. Enyashin,[9,10] Lothar Houben,[4*] and Reshef Tenne[1*]

(Dedicated to Professor John B. Goodenough on the Occasion of his 100th year Birthday. Until hundred and twenty like twenty (a proverb in Hebrew)

[1]Department of Molecular Chemistry and Materials Science, Weizmann Institute of Science, Rehovot 7610001, Israel
[2]CEITEC – Central European Institute of Technology, Brno University of Technology, Purkyňova 123, 612 00 Brno, Czech Republic
[3]Deutsches Elektronen-Synchrotron DESY, Notkestr. 85, 22607 Hamburg, Germany
[4]Department of Chemical Research Support, Weizmann Institute, Rehovot 7610001, Israel
[5]Ames Laboratory, U.S. Department of Energy, Ames, IA, 50011-3020, USA
[6]ProChem, Inc. 826 Roosevelt Road Rockford, IL 61109
[7]Department of Physics, SUNY Buffalo State, Buffalo, New York 14222, USA
[8]Institute of Physical Engineering, Brno University of Technology, Technická 2, 616 69 Brno, Czech Republic
[9]Institute of Solid State Chemistry UB RAS, 620990 Ekaterinburg, Russian Federation
[10]Ural Federal University, Institute of Natural Sciences and Mathematics, 620083 Ekaterinburg, Russian Federation

[*]corresponding authors- reshef.tenne@weizmann.ac.il, lothar.houben@weizmann.ac.il, kolibal.m@fme.vutbr.cz

This file includes:

SI text
SI Figures S1-S14
SI References 1-5

SI text:
Characterization details

Scanning electron microscopy (SEM)

SEM imaging of the nanotubes was done with a Zeiss Sigma 500 model. A minute quantity of native powder sample was picked up by a capillary tube and spread over carbon tape for the SEM analysis. Energy-dispersive X-ray spectroscopy analysis (EDS) was performed with the Bruker XFlash/60mm retractable detector installed in the Zeiss Sigma SEM. The quantification of the chemical elements is based on standard-less and self-calibrating spectrum analysis, using the ZAF matrix correction formulas. The relative abundance (yield) of the NT was estimated by analyzing many SEM images of the product. ImageJ software[1] has been used for the analysis of the nanotubes' abundance and their size distribution. The determined abundancies were based on counting the number of nanotubes and flakes in each image and dividing the number of nanotubes by the total number of nanotubes and flakes. Similarly, the diameter of the nanotubes (> 100 tubes in each case) was measured using ImageJ software by calibrating the scale in the image. The abundance of nanotubes (number of nanotubes with a given diameter in the total number of nanotubes analyzed) was plotted as a function of the diameter. While being only semi-quantitative in nature, the overall yield did not vary appreciably from one batch to the other.

X-ray powder diffraction

X-ray powder diffraction (XRD) measurements were performed using TTRAX III (Rigaku, Tokyo, Japan) theta-theta diffractometer. The set-up was equipped with a rotating copper anode X-ray tube operating at 50 kV/200 mA. The powders were spread on a zero-background Si holder and pressed with glass to flatten the surface. A bent graphite monochromator and a scintillation detector were aligned to the diffracted X-ray beam. They were scanned in specular diffraction ($\vartheta/2\vartheta$ scans) from 3-80° (2ϑ) with a step size of 0.02° and a scan rate of 0.5° per min in Bragg-Brentano mode with variable slits. The XRD data was analyzed using JADE Pro software and PDF-4+ 2020 database (ICDD).

Transmission electron microscopy, STEM-EDS and EEL Spectroscopy

Transmission electron microscopy (TEM) and selected area electron diffraction (SAED) patterns analyses were performed using a JEOL JEM2100 microscope operated at 200 kV. The analysis of the TEM images, including intensity profiles along the *c*-axis, and the SAED has been performed with Digital Micrograph 3.1.0 (Gatan) software.

A double aberration-corrected Titan Themis Z microscope (Thermo Fisher Scientific (TFS) Electron Microscopy Solutions, Hillsboro, USA) equipped with a high-brightness field emission gun and a Wiener-type monochromator was employed for the atomic resolution HR-STEM imaging and monochromated EEL spectroscopy at an accelerating voltage of 200 kV. HAADF-STEM images were recorded with a Fischione Model 3000 detector with a semi-convergence angle of 21.4 mrad, a probe current of 40 pA, and an inner collection angle of 70 mrad. Large angle bright-field images were taken with a TFS BF STEM detector with an outer collection angle of 18 mrad. EDS hyperspectral maps were collected with a SuperX G2 four-segment SDD detector with a probe semi-convergence angle of 21 mrad, a beam current of approximately 200 pA, a pixel dwell time of 10-20 µs and a total recording time of typically 10 minutes. Quantitative maps were analyzed

with the TFS Velox software, through background subtraction and spectrum deconvolution. A correction of frame-to-frame beam/specimen drift was employed where required using custom software in order to refine net intensity profiles. Monochromated EEL spectra were recorded at a system energy resolution of 80 meV in Dual-EEL spectrum mode on a Gatan Quantum GIF 966ERS energy loss spectrometer (Gatan Inc., Pleasanton, USA) with an Ultrascan1000 CCD camera. The EEL spectra were recorded with a STEM probe with a semi-convergence angle of 24 mrad and a beam current of 200 pA by summing multiple 2 ms spectrum acquisitions from a spectrum image map taken over a larger field of view to distribute the electron exposure. The outer semi-collection angle of the spectrometer was 50 mrad. DigitalMicrograph (Gatan Inc., Pleasanton, USA) was used for the quantification of the chemical shift of the La M core loss from the monochromated EEL spectra. In all cases of atomic-resolution HRSTEM analyses prior specimen cleaning steps, e.g. plasma cleaning, were avoided in order to preserve the surface structure of the nanostructures.

Preparation of nanotube cross-section lamella with focused ion beam (FIB)

A cross-section lamella of $(SmS)_{1.19}TaS_2$ nanotube was prepared using FIB-SEM Helios 660 Dual Beam microscope (Thermo Fisher Scientific (TFS) Electron Microscopy, Hillsboro, USA) equipped with Ga ion source $(SmS)_{1.19}TaS_2$ was dispersed in ethanol, drop cast on Si(100) substrate, and the sample was dried under normal conditions The lamella was prepared by a standard lift-out process using carbon EBID (50 nm) followed by carbon IBID (2 µm) deposited sequentially with stage tilt ±20° to minimize gaps between the protective layer and the nanotube. Final polishing was done at 2 kV ion beam energy. As prepared lamellae of a nanotube cross-sections were further inspected using Talos FX 200 and Titan-Themis Z double corrected transmission electron microscope.

X-ray photoelectron spectroscopy (XPS)

The powder samples were prepared in a glove box, under N_2 atmosphere, and put on a carbon tape such as to get a dense yet very thin layer of grains, intentionally a monolayer of grains. Then, sample transfer to the vacuum chamber involved exposure to ambient for only a fraction of a minute. Base pressure below $1\cdot10^{-9}$ torr was kept in the analysis chamber. The XPS measurements were performed on a Kratos AXIS Ultra DLD spectrometer, using a monochromatic Al kα source at 15-75 W and detection pass energies of 20-80 eV. In-situ work-function measurements on the 'as received' samples were conducted under extremely low power of the X-ray source, 0.2 W.

In order to eliminate charging effects, measurements under both positive and negative charging conditions were compared, yielding no observable line shifts of the major lines (yet, some of the oxidized components underwent small shifts, in the range of 70-200 meV). Consequently, for both types of samples, all line-shape analyses relevant to the discussion in this report were practically free of charging-related artefacts. Complementarily repeated scans on given spots were conducted to identify potential beam-induced sample damage during extended exposures to the X-ray irradiation. No observable damage signatures were found.

Curve fitting of the leading signals in the misfit nanotubes yields two components of 'perfect' constituents, SmS and TaS_2, with stoichiometries very close to the theoretical ones, however with an additional component of partially oxidized $TaS_{2-x}O_x$. The latter component is associated with

surface imperfections, to which XPS presents enhanced sensitivity. Representative atomic concentration ratios are given in **Table S1**. Note the slight deviation of S/(2Ta+Sm) from the ideal value of unity. Also, note that Sm/Ta = 1.12 is slightly lower than the ideal 1.19 value. Both latter deviations from perfectly stoichiometric ratios are associated with small amounts of oxidized Ta (Ta^4) that could be resolved within the Ta 4f spectral window. Once taken into account, the related concentration ratios become very close to the ideal values, as given in brackets in **Table S1**.

Table S1. Atomic concentration ratios in the misfit nanotubes, as derived from the XPS curve fitting. S^1 and Ta^1 refer to the TaS_2-related core-level components at, respectively, 161.04 eV and 22.88 eV. S^2 is the SmS-related component at 161.69 eV. S^3 and Ta^2 appear, respectively, at 162.25 eV and 23.53 eV. Correction to the Ta 4f evaluation due to overlapping O 2s signal was applied by referencing to the O 1s signal (not shown here). Values in brackets correspond to calculations in which the Ta^4 component is excluded.

	S^1/Ta^1	S^2/Sm	S^3/Ta^2	Sm/Ta	S/(2Ta+Sm)
XPS-derived	1.99	0.95	1.53 (2.04)	1.125	0.91 (0.98)
theoretical	2.0	1.0	- - -	1.19	1.0

X-ray absorption spectroscopy (XAS): Sample preparation and measurements

Samples were mixed with cellulose binder (mixing ratio 1:3) and pressed with the hydraulic press (pressure: 2 ton) to produce 10 mm pellets suitable for analysis in transmission geometry. Mixing and handling of the powders were performed inside the Ar-filled glove box (H_2O and O_2 concentration < 1 ppm) to avoid possible oxidation of the samples. Before analysis, the sample pellets were placed between two Kapton foils (thickness: 50 micron) inside a special sample holder forming a closed volume to save from atmospheric air.

XAS experiments in transmission mode were performed at DESY P23 "In-situ and X-ray imaging beamline". The experimental set-up consisted of entrance slits, the first X-ray detector, a rotating sample stage, and a second X-ray detector. Liquid N_2 cooled Double Crystal Monochromator (Si 111) was used for choosing the required energy, X-ray mirrors with B_4C or Rh coating were used for harmonic rejection depending on the edge. Silicon avalanche photodiodes (APD) were used for measuring the intensity of incoming and transmitted X-ray beam. The sample pellet was mounted on the OWIS DRTM 40 rotary stage and rotated with a speed of 180°/sec during the analysis.

In accordance with the proposed setup, X-ray absorption of the sample was measured at different X-ray energies in the vicinity of Ta L_3 and Sm L_3 X-ray absorption edges. The X-ray absorption of the sample at a certain energy point was measured for 10 sec. The energy of the incoming X-ray was also corrected by measuring the absorption spectra of pure Ta (99,99 %, L-III edge) and Mn (99,99 %, K-edge) foils. APD detector data were corrected for a dead time,[2] EXAFS spectra processing and analysis were done by Larch,[3] Athena and Artemis software.[4]

SI Figures:

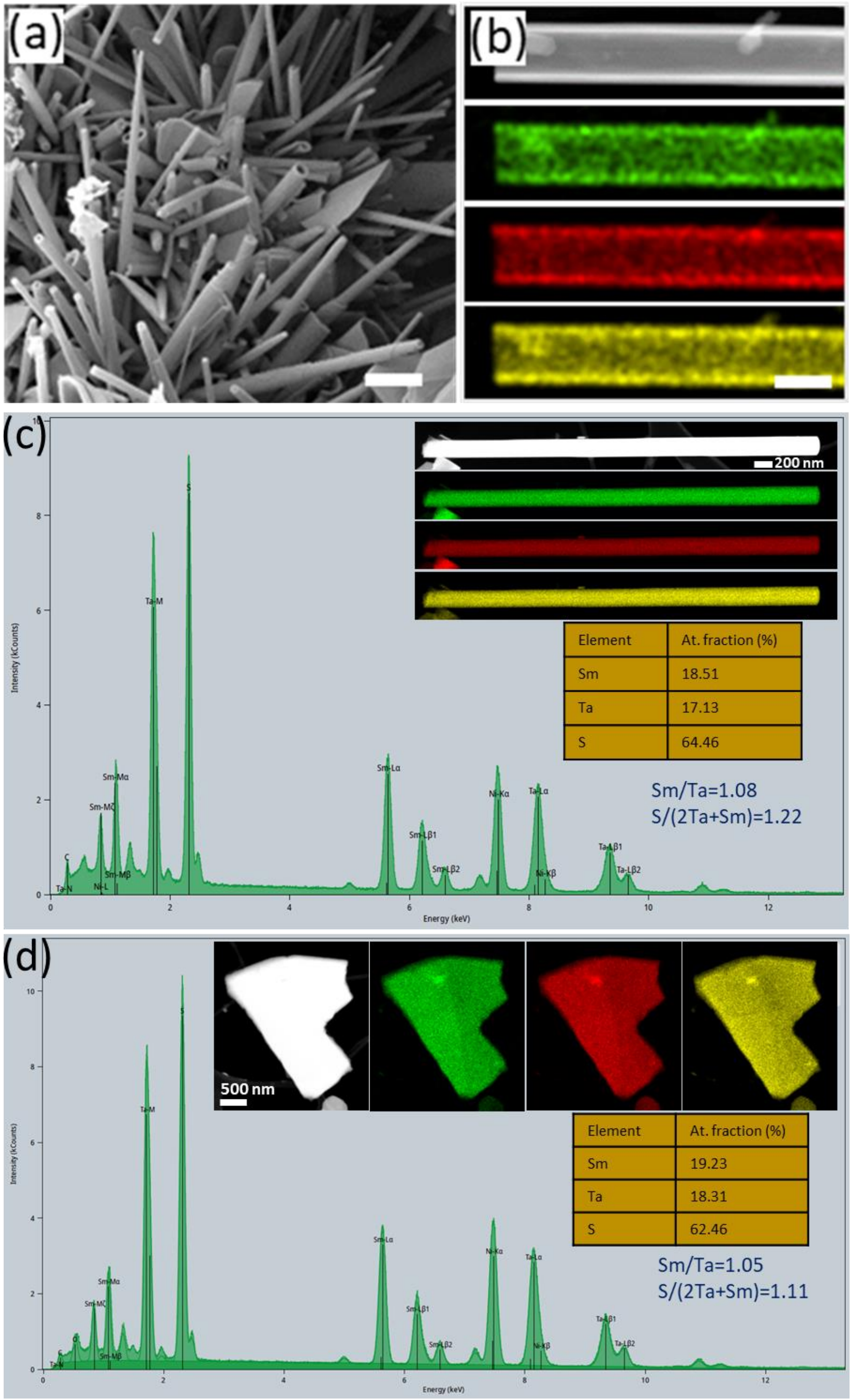

Fig. S1*. (a) SEM micrographs of $(SmS)_{1.19}TaS_2$ tubular structures and commonly observed MLC flakes and nanoscrolls obtained by CVT reaction, scale bar is 2 µm. (b) STEM HAADF images of a single $(SmS)_{1.19}TaS_2$ MLC nanotube and corresponding SEM-EDS chemical maps (Sm-red, Ta-green and S- yellow), scale bar 200 nm. (c and d) STEM-EDS spectrum and chemical maps (inset) of individual nanotube and flake of $(SmS)_{1.19}TaS_2$ obtained on a nickel grid. The table in the insets show the atomic fraction of the elements.*

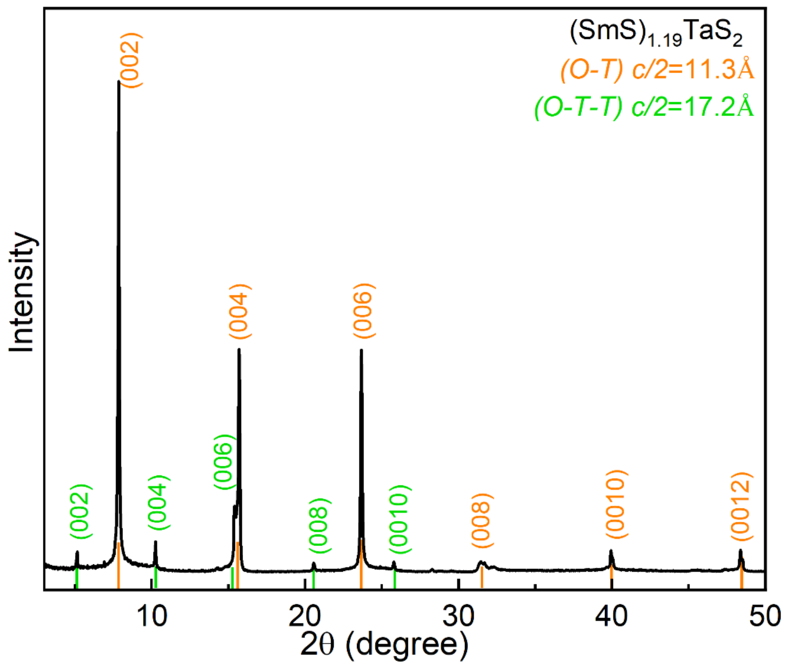

Fig. S2*. *X-ray powder diffraction analysis: *Representative XRD patterns of the $(SmS)_{1.19}TaS_2$ MLC product (flakes + nanotubes). The highly preferred orientation of (00l) Bragg planes associated with the (O-T) with c-axis periodicity 11.3 Å is represented with solid orange lines. The additional (much weaker) periodicity of 17.2 Å corresponds to the period doubling of TaS_2 i.e. (O-T-T).*

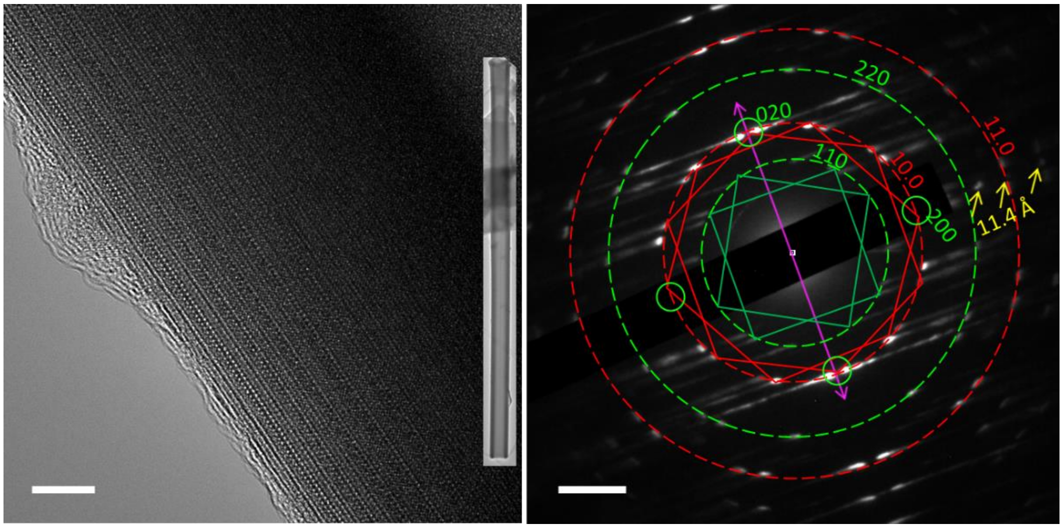

Fig. S3. *(a) High magnification TEM image of $(SmS)_{1.19}TaS_2$ nanotube, the periodic stacking SmS and TaS_2 subunits of MLC structure is revealed, scale bar- 5 nm. A low magnification image of the corresponding nanotube (diameter 240 nm) is presented in the inset. (b) SAED patterns collected from the nanotube in (a), the set of diffraction spots corresponding to SmS and TaS_2 were identified with green and red dotted circles, respectively. The corresponding Miller indices were marked. Small yellow arrows indicate the basal reflections and the tubule axis is marked by purple double arrows. The two sets of four pairs (110) reflections of the SmS are marked by rotated green squares. The sets of six pairs (10.0) reflections of the TaS_2 are marked by red hexagons. These sets of reflections are rotated by 30° with respect to each other. Here crystallographic b axis (i.e. 020) coincides with the nanotube growth axis. The splitting of the spots is due to the chiral angle of the nanotube.*

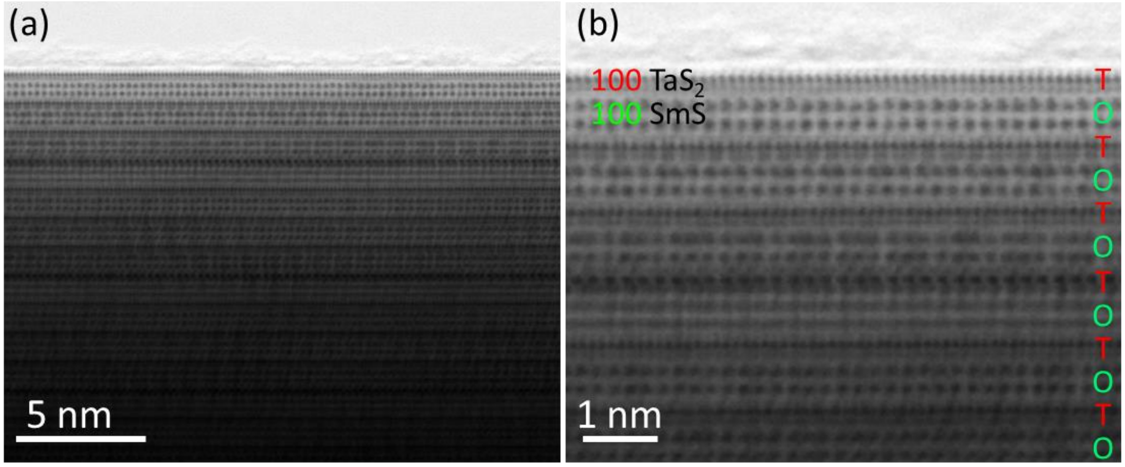

Fig. S4. *(a) High-magnification STEM-BF images of a portion of $(SmS)_{1.19}TaS_2$ nanotube, and (b) a magnified image revealing atomic coordination. The layers are stacked alternately along the c-axis. Projections of distorted rocksalt SmS and hexagonal TaS_2 layers were marked in green and red colors.*

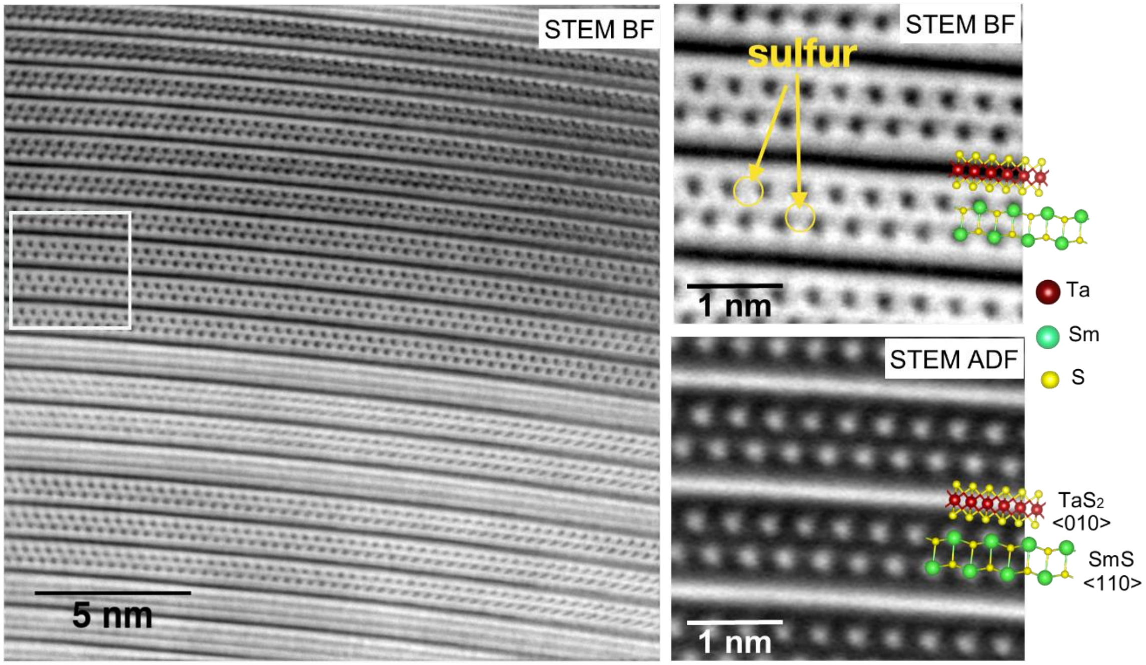

Fig. S5. *Atomic resolution HR-STEM and the corresponding magnified BF and ADF image of a portion of a $(SmS)_{1.19}TaS_2$ nanotube lamella. The S atoms in the distorted rocksalt layers of SmS are clearly visible.*

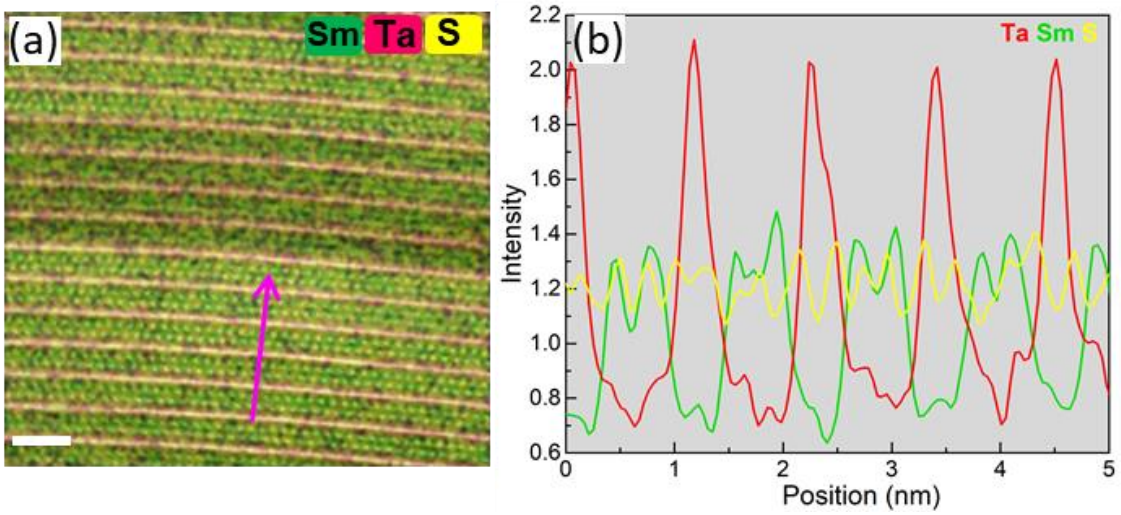

Fig. S6. *HR-STEM-EDS chemical maps of* $(SmS)_{1.19}TaS_2$ *nanotube lamella (a) The elemental distribution maps of Sm (green), Ta (red) and S (yellow), confirm the antiphase relationships between the Sm-Ta, scale bar 2 nm. (b) STEM-EDS net intensity profiles of the elements drawn from a region marked in the pink arrow of (a). The S profile clearly reveal its coordination with Ta and Sm atoms. The sulfur atomic layers either side of the Ta layer is evident from the displayed profile.*

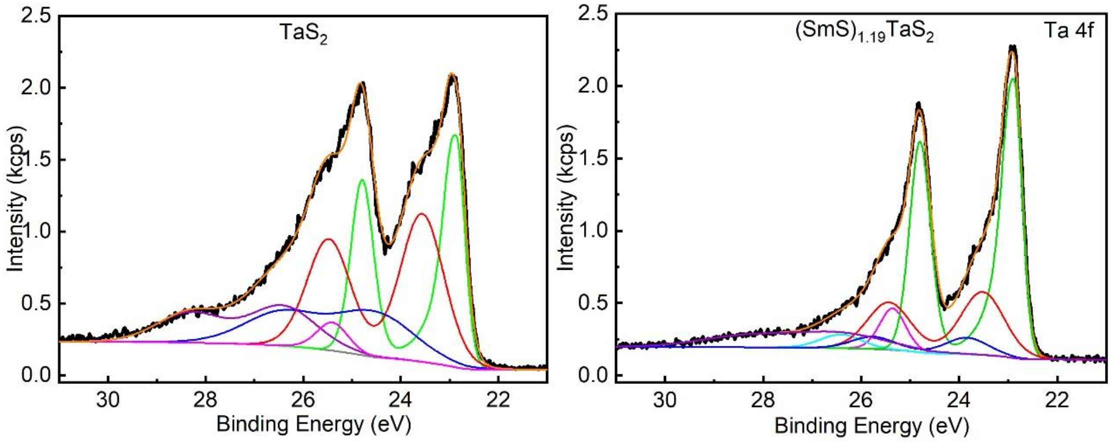

Fig. S7. *Curve fitting of the XPS Ta 4f core-level in (a) pristine* TaS_2 *and (b)* $(SmS)_{1.19}TaS_2$ *misfit compound. Green curves are attributed to the TaS2 layers. Enhanced Ta oxidation in* TaS_2 *(panel a, brown and blue curves) is observed, attributed to open platelet edges. Note that the O 2s level is seen as well in this energy window, peaking around 25.4 eV.*

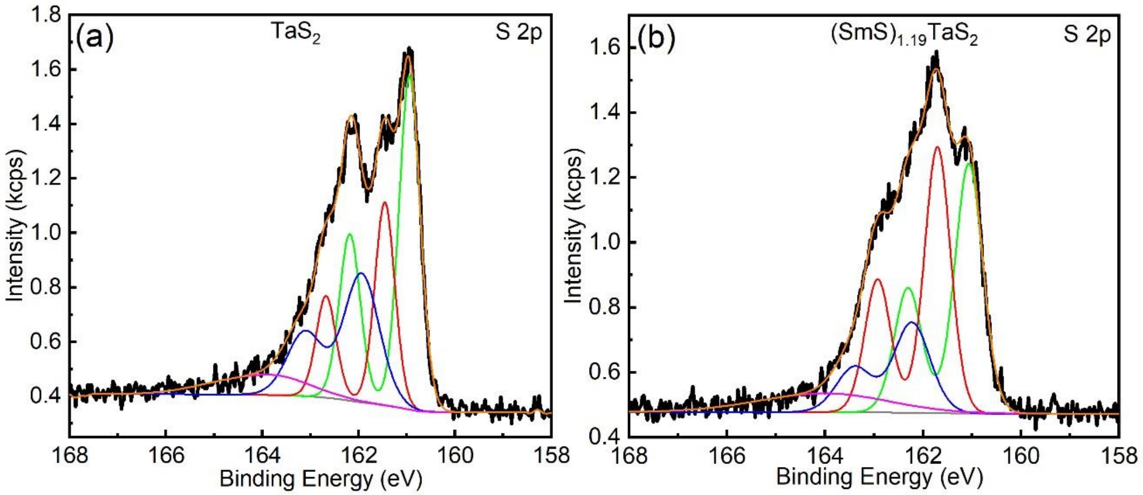

Fig. S8. *Curve fitting of the XPS S 2p core-level in (a) pristine TaS_2 and (b) $(SmS)_{1.19}TaS_2$ misfit compound. Green curves are associated with the TaS_2 layers. The brown curve in panel b, with S $2p_{3/2}$ peak at 161.7 eV, is attributed to the SmS constituent. In contrast, the brown curve in panel a, with a corresponding peak at 161.45 eV, is attributed to S atoms in the vicinity of Ta-O bonds. Additional oxidation states of S, mainly expressed by the blue curves, indicate on some inhomogeneity in both samples.*

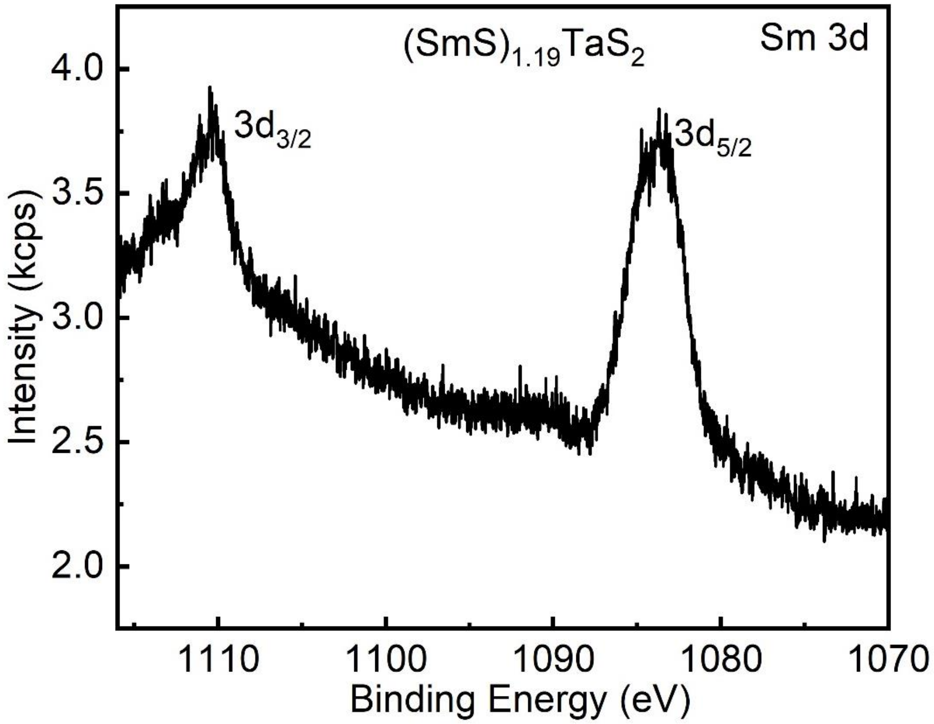

Fig. S9. *XPS of Sm 3d core levels in $(SmS)_{1.19}TaS_2$ misfit compound. The binding energy of Sm 3d matches a Sm^{3+} oxidation state, which indicates charge transfer from SmS to TaS_2.*

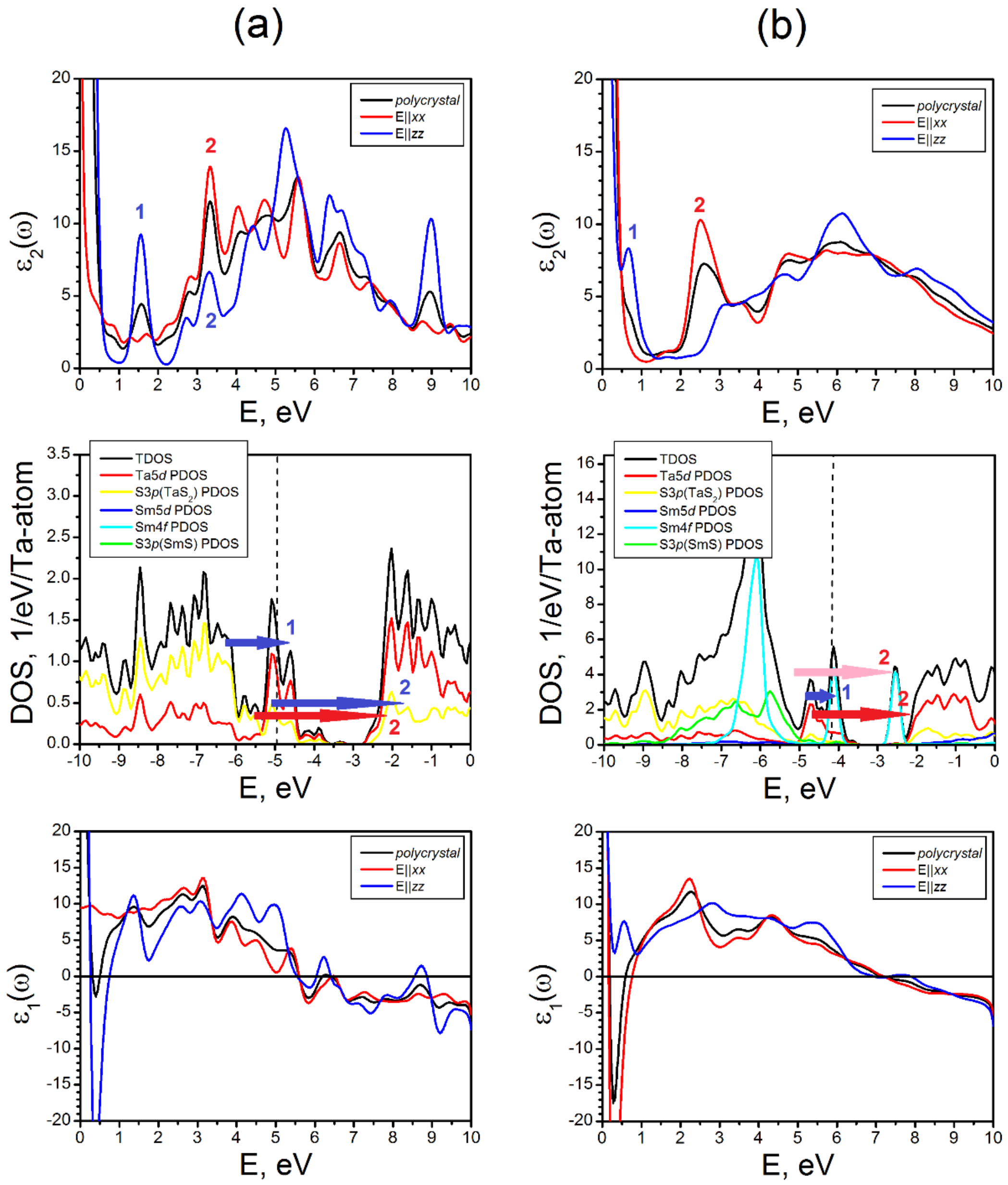

Fig. S10. *Imaginary (ε_2) and real (ε_1) part of the frequency-dependent dielectric function, obtained from DFT LDA+U calculations. The graphs display the anisotropic dielectric response depending on the polarization of the electric field. The z-axis coincides with c-axis of the crystal, x with an in-plane orientation in the basal plane. 'Polycrystal' refers to an average over three spatial directions. (a) Bulk 2H-TaS_2, (b) bulk misfit compound $(SmS)_{1.20}TaS_2$. The DOS picture is employed to unveil the possible origin of the most prominent maxima in the imaginary part. Particularly, the strong peak in the imaginary part of the dielectric function of TaS_2 is related to transitions into the half-filled Ta5dz^2-band (peak 1 in (a)) (see also ref [5]). This peak is absent in the misfit compound due to charge transfer from SmS to TaS_2 and filling of the Ta5dz^2-band.*

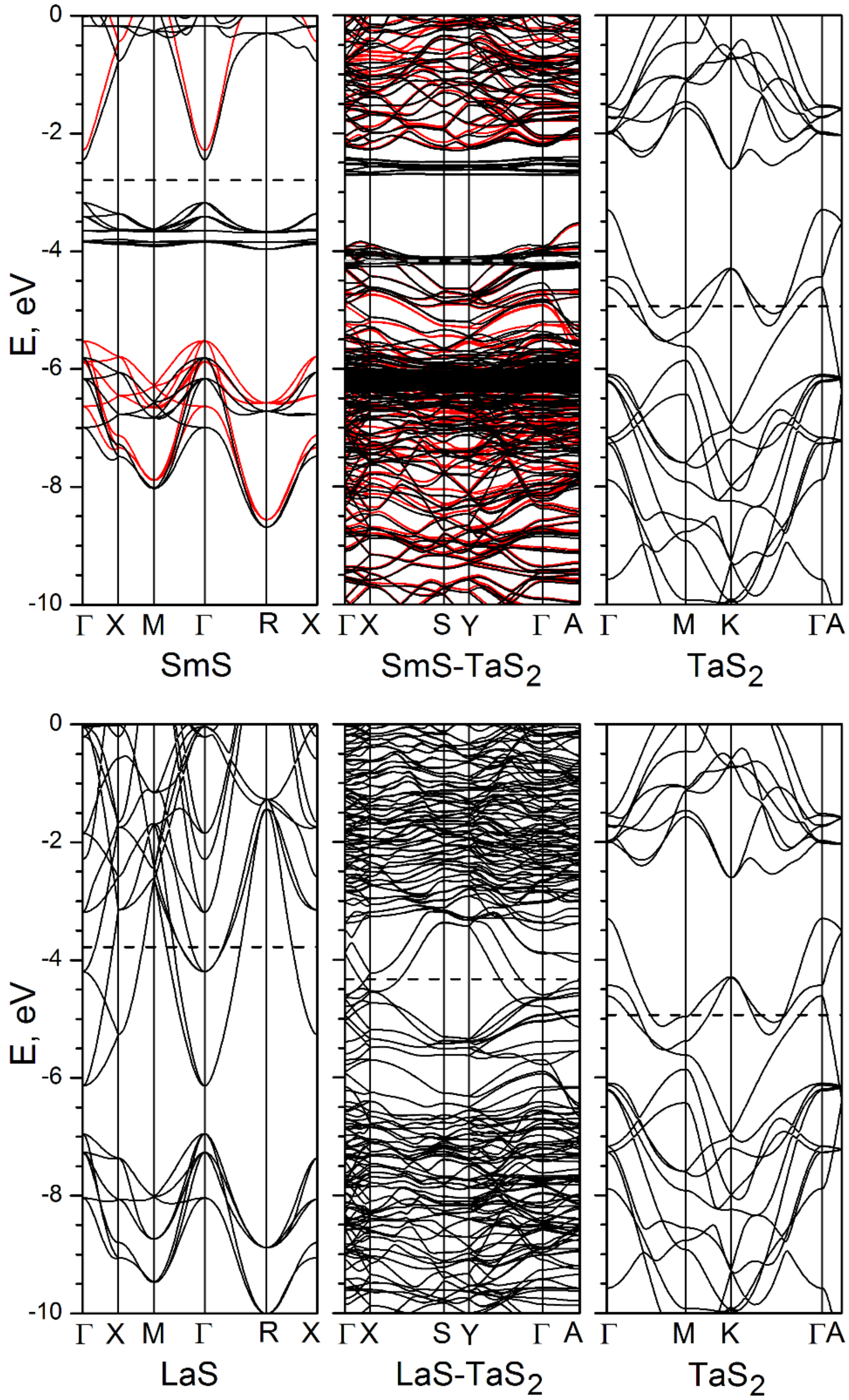

Fig. S11. *Band structure for the bulk rare-earth monosulfides fcc-SmS; fcc-LaS, the bulk 2H-*TaS_2 *and their misfits with compositions* $(SmS)_{1.20}TaS_2$ *and* $(LaS)_{1.20}TaS_2$*. The bands with different spin-polarization are drawn as black and red lines. Fermi level is marked by a dashed line. DFT LDA+U calculations.*

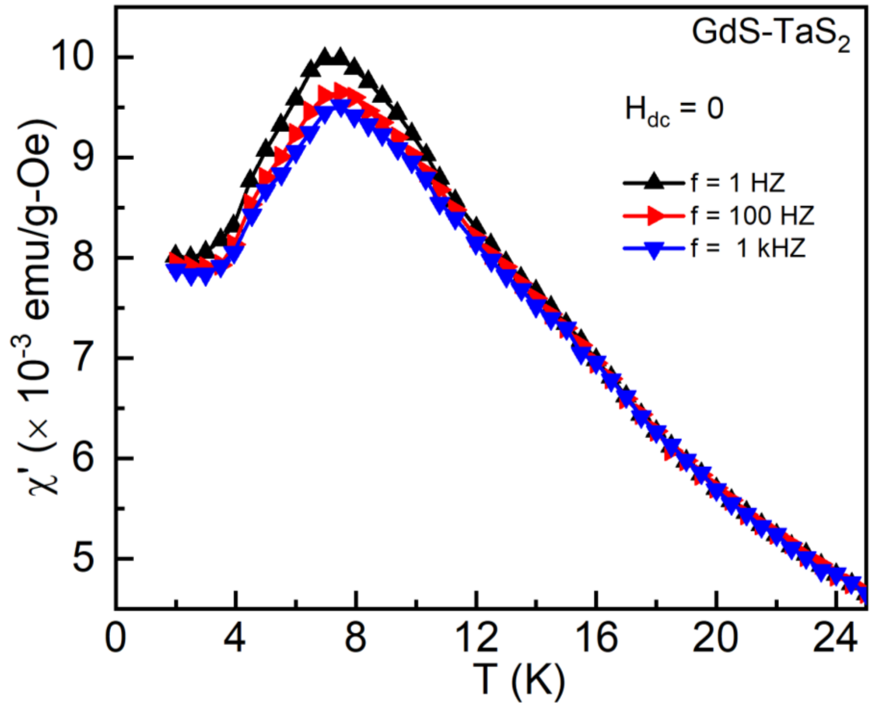

Fig. S12. *Temperature-dependent magnetic susceptibility of GdS-TaS_2 MLC, (a) Real part of the ac magnetic susceptibility measured at frequencies, f = 1, 100 and 1000 Hz and H_{dc} = 0.*

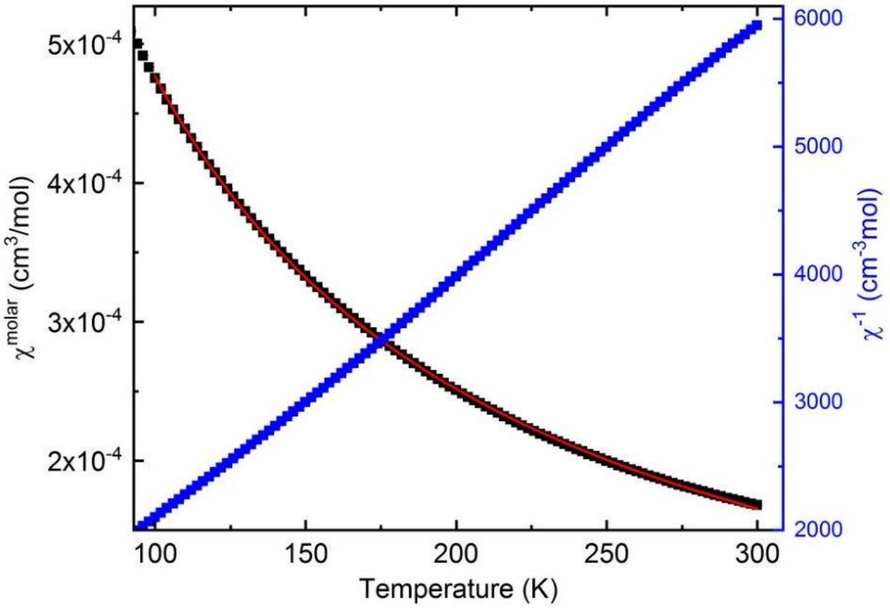

Fig. S13*. Temperature dependence of magnetic susceptibility of $(SmS)_{1.19}TaS_2$ MLC measured at 5000 Oe shown as $\chi(T)$ and also as $\chi^{-1}(T)$. Fitting of $\chi(T)$ by Curie-Weiss equation is shown by the red line.*

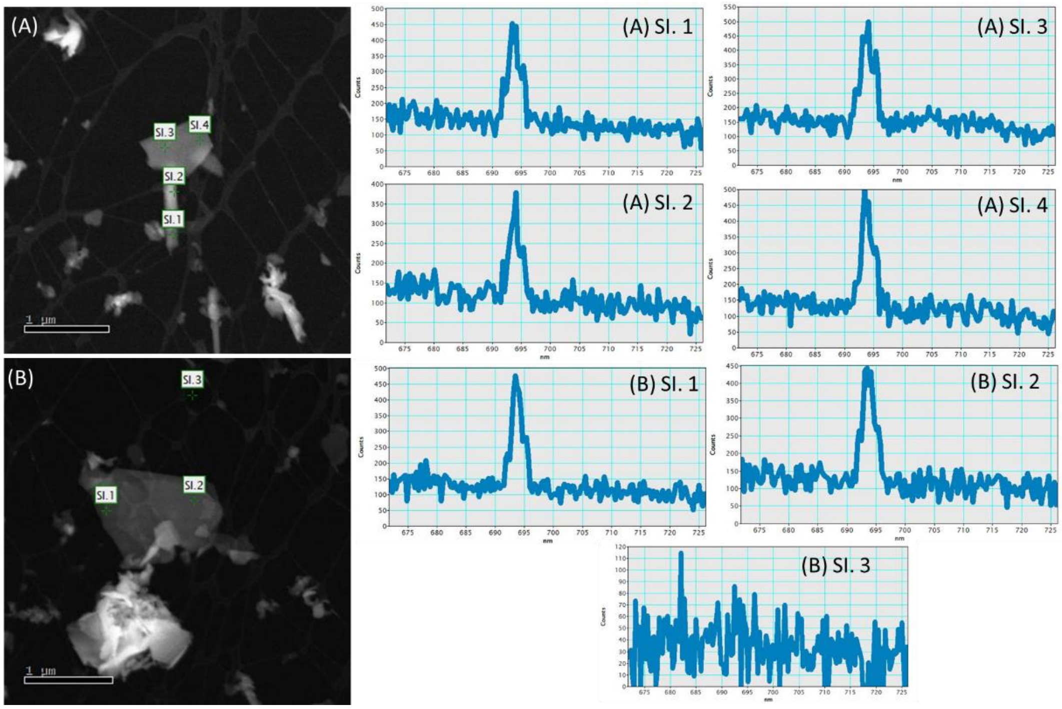

***Fig. S14**. Cathodoluminescence measurements of individual nanotubes and flakes of $(SmS)_{1.19}TaS_2$ at cryogenic temperatures (-150 K) carried-out within the SEM. The SEM parameters were set to 20 kV, aperture 120µm, high current and analytic mode. The CL spectra in right panel were collected from the marked regions in the SEM image. Each spectrum was measured separately, one by one. Pixel size is 54nm. Spectrum was collected for 20 seconds for every pixel. A 1200 lines/mm grating was used with a slit size of 1mm (2nm spectral resolution). The spectra collected on MLC nanotube/flake show a peak around 695 nm while spectrum collected at the background (B SI. 3) does not show any peak, which confirms the emission is inherently from the nanotube/flakes.*

SI References:

1. Schneider, C. A.; Rasband, W. S.; Eliceiri, K. W., NIH Image to ImageJ: 25 years of image analysis. *Nature Methods* **2012,** 9, (7), 671-675.

2. Kishimoto, S., Behavior of a fast counting system with an avalanche photodiode detector for synchrotron X-rays. *Nucl. Instruments Methods Phys. Res. Sect. A Accel. Spectrometers, Detect. Assoc. Equip.* **1997,** 397, (2), 343-353.

3. Newville, M., Larch: An Analysis Package for XAFS and Related Spectroscopies. *J. Phys. Conf. Ser.* **2013,** 430, 012007.

4. Ravel, B.; Newville, M., ATHENA, ARTEMIS, HEPHAESTUS: data analysis for X-ray absorption spectroscopy using IFEFFIT. *J. Synchrotron Radiat.* **2005,** 12, (Pt 4), 537-41.

5. Yan-Bin, Q.; Yan-Ling, L.; Guo-Hua, Z.; Zhi, Z.; Xiao-Ying, Q., Anisotropic properties of TaS_2. *Chinese Phys.* **2007,** 16, (12), 3809-3814.
